# Protection against fibrosis by a bacterial consortium in metabolic dysfunction-associated steatohepatitis and the role of amino acid metabolism

**DOI:** 10.1080/19490976.2024.2399260

**Published:** 2024-09-06

**Authors:** Suet-Ying Kwan, Kristyn A. Gonzales, Mohamed A. Jamal, Heather L. Stevenson, Lin Tan, Philip L. Lorenzi, P. Andrew Futreal, Ernest T. Hawk, Joseph B. McCormick, Susan P. Fisher-Hoch, Robert R. Jenq, Laura Beretta

**Affiliations:** a Department of Molecular and Cellular Oncology, The University of Texas MD Anderson Cancer Center, Houston, TX, USA; b Department of Genomic Medicine, The University of Texas MD Anderson Cancer Center, Houston, TX, USA; c Department of Pathology, The University of Texas Medical Branch, Galveston, TX, USA; d Metabolomics Core Facility, Department of Bioinformatics and Computational Biology, The University of Texas MD Anderson Cancer Center, Houston, TX, USA; e Department of Clinical Cancer Prevention, The University of Texas MD Anderson Cancer Center, Houston, TX, USA; f School of Public Health, University of Texas Health Science Center at Houston, Brownsville, TX, USA

**Keywords:** Liver fibrosis, gut microbiota, probiotics, amino acids, bacterial consortium

## Abstract

The gut microbiota drives progression to liver fibrosis, the main determinant of mortality in metabolic dysfunction-associated steatohepatitis (MASH). In this study, we aimed to identify bacterial species associated with protection against liver fibrosis in a high-risk population, and test their potential to protect against liver fibrosis *in vivo*. Based on stool shotgun metagenomic sequencing of 340 subjects from a population cohort disproportionally affected by MASH, we identified bacterial species from the *Bacteroidales* and *Clostridiales* orders associated with reduced risk of liver fibrosis. A bacterial consortium was subsequently tested in a mouse model of MASH, which demonstrated protective effects against liver fibrosis. Six of the eight inoculated bacteria were detected in mouse stool and liver. Intrahepatic presence of bacteria was further confirmed by bacterial culture of mouse liver tissue. Changes in liver histological parameters, gut functional profiles, and amino acid profiles were additionally assessed. Comparison between fibrosis-associated human metagenome and bacteria-induced metagenome changes in mice identified microbial functions likely to mediate the protective effect against liver fibrosis. Amino acid profiling confirmed an increase in cysteine synthase activity, associated with reduced fibrosis. Other microbiota-induced changes in amino acids associated with reduced fibrosis included increased gut asparaginase activity and decreased hepatic tryptophan-to-kynurenine conversion. This human-to-mouse study identified bacterial species and their effects on amino acid metabolism as innovative strategies to protect against liver fibrosis in MASH.

## Introduction

Metabolic dysfunction-associated steatotic liver disease (MASLD) affects 25% of the adult population and ranges from simple steatosis to metabolic dysfunction-associated steatohepatitis (MASH). Patients with steatohepatitis can further develop liver fibrosis, cirrhosis, and hepatocellular carcinoma (HCC). The presence of liver fibrosis is the most important determinant of mortality in MASLD patients.^1,2^

Animal studies have demonstrated a causative role for the gut microbiota in MASLD development and progression.^3–5^ In human cross-sectional studies, specific taxonomic changes have been associated with the development of steatohepatitis,^6^ fibrosis,^7^ cirrhosis,^8^ and progression to HCC.^9,10^ Modulation of the gut microbiota using antibiotics, probiotics, prebiotics or synbiotics, has therefore been investigated as a potential strategy for MASLD treatment.^11^ However, clinical studies using synbiotics or probiotics have shown only mixed results in improvement of liver fibrosis.^12–16^ These studies used strains predominantly belonging to the *Lactobacillus* and *Bifidobacterium* genera. While these are the most commonly used commercial probiotics, other species have arisen as potential “next-generation” probiotics.^17^ The use of more relevant strains with observed associations with liver fibrosis in a human population, may have greater benefit in the prevention or treatment of liver fibrosis. We recently reported changes in gut microbiota associated with liver fibrosis in 217 subjects randomly selected from a population-based cohort disproportionally affected by obesity (51%), diabetes (28%), MASLD (52%), and liver fibrosis (14%).^18^ In this study, we applied shotgun metagenomic sequencing instead of 16S rRNA sequencing, extended the study to a larger set of 340 subjects from the cohort, and focused on depleted bacterial species to test their potential to protect against liver fibrosis *in vivo*. We also performed comparative human and mouse metagenomic functional analysis to identify mechanisms of action of the bacterial consortium-induced protective effect against liver fibrosis.

## Materials and methods

### Study participants

This study includes 340 participants randomly selected from the Cameron County Hispanic Cohort, a population-based cohort of Mexican Americans in South Texas.^19^ Participants had clinical visits and stool collected between February 2018 and August 2021. Subjects positive for hepatitis B or C viruses, or those who had antibiotic, probiotic, or proton pump inhibitor use within 30 days, were excluded. Demographic and clinical parameters are shown in Supplemental Table S1. Written informed consent was obtained from each participant, and the study protocol was approved by the Institutional Review Board at the University of Texas MD Anderson Cancer Center (approved protocol number PA13–0317). Using vibration-controlled transient elastography (VCTE) (FibroScan® 502 Touch or FibroScan® 530 Compact, Echosens), trained operators obtained CAP measurements (dB/m) for detection of steatosis, and liver stiffness measurements (LSM, in kiloPascals, kPa) for liver fibrosis. Based on the literature and AASLD Practice Guidance,^20,21^ liver steatosis was defined as CAP ≥ 268. LSM < 6.2 was used to rule out significant liver fibrosis (F2-F4), while significant liver fibrosis was defined as LSM ≥ 7.3 kPa. LSM < 8.0 was used to rule out advanced liver fibrosis (F3-F4), while advanced fibrosis was defined as LSM ≥ 8.8 kPa. LSM measurements were considered inconclusive with <10 valid measures or interquartile range-to-median ratio >0.3. Stool samples were collected using the OMNIgene® GUT stool collection kit (DNA Genotek, Ontario, Canada).

### Bacterial strains selection

To design a bacterial consortium relevant to the human data for the *in vivo* study, we used for each species, the most abundant strain in the human cohort data that was also commercially available (Supplemental Table S6). *Parabacteroides distasonis* (ATCC 8503), *Anaerobutyricum hallii* (ATCC 27751), *Gemmiger formicilis* (ATCC 27749), *Bacteroides caccae* (ATCC 43185), *Bacteroides ovatus* (ATCC 8483), and *Bacteroides uniformi*s (ATCC 8492) were purchased from the American Type Culture Collection (ATCC). *Bacteroides finegoldii* (DSM 17565) and *Alistipes onderdonkii subsp. vulgaris* (DSM 108977) were purchased from the Leibniz-Institute DSMZ-German Collection of Microorganisms and Cell Cultures.

To determine the relevance of the six colonizing species in the consortium to our human cohort, we also retrieved the whole-genome sequences for all strains detected in the human cohort for these six species (Supplemental Table S6). Raw genome sequencing files were downloaded from the NCBI data hub (https://www.ncbi.nlm.nih.gov/). Each raw sequencing file was annotated using the prokka pipeline [v1.14.16] available on the Proksee online tool (https://proksee.ca/). This confirmed that the presence of cysteine synthase was universal across all strains interrogated. On the contrary, serine acetyltransferase was only present in *Bacteroides uniformis* ATCC 8492 used in the *in vivo* study, and additional rare strains [*Bacteroides uniformis* strain (dnLKV2) and *Bacteroides ovatus* strains (3_8_47FAA, str. 3725 D1 iv, str. 3725 D9 iii)]. The presence of the asparaginase gene was universal across all strains detected in the human cohort.

### Preparation of the bacterial consortium

Each bacterial strain was grown as monocultures in a Whitley A45 anaerobic workstation (10% H_2_, 5% CO_2_, and 85% N_2_), on trypticase soy agar, Brucella agar, Columbia agar, or BYEM10+mucin agar.^22^ Prior to each preparation of inoculum, the identity of each bacterial strain was confirmed by biotyping using a BRUKER Matrix-Assisted Laser Desorption/Ionization Time-Of-Flight (MALDI-TOF) mass spectrometry. Bacterial cells were resuspended in phosphate-buffered saline (PBS), quantified using a Nexcelom Cellometer cell counter with Syto BC green and propidium iodide staining, and combined for the preparation of inoculum containing 5 × 10^8^ colony-forming units (CFU) of each strain/ml. An aliquot of each preparation was taken prior to inoculation, and the bacterial pellet was stored at −80°C until sequenced.

### Bacterial inoculation and diet-induced steatohepatitis of germ-free mice

The methionine- and choline-deficient (MCD) diet was used to induce liver fibrosis in germ-free (GF) mice. Eight-week-old male, GF C57BL/6J mice were purchased and housed at the Germ-Free Facility of the Baylor College of Medicine Gnotobiotics Core, under standard conditions of temperature, humidity, and light control. Mice were group-housed with one flexible film isolator used per experimental group. All technical manipulations were conducted by facility staff blinded to the groups while maintaining GF and gnotobiotic conditions. Mice were randomized to control and treatment groups. Animal procedures were carried out in accordance with the policies and regulations of the Institutional Animal Care and Use Committee at Baylor College of Medicine. As consecutive inoculations of bacteria improves colonization in GF mice,^23^ we inoculated mice with the bacterial consortium three times. On days 0, 5, and 10, mice in the control group (GF-MCD) (*n* = 11) were administered vehicle control (PBS), while mice in the treated group (GF-MCD-B) (*n* = 10) were inoculated by oral gavage with a pool containing 1 × 10^8^ CFU of each of the eight bacterial strains (200 µl total volume). On day 14, mice were switched from regular chow (autoclaved Select Rodent Diet 50 IF/6F, #5V0F, LabDiet; Supplemental Table S2) to MCD (#A02082002BR from Research Diets, double irradiated, with 1.5× vitamins; Supplemental Table S2).^24^ Stool was collected on days 0, 14, and 28. Mice were sacrificed by CO_2_ asphyxiation, followed by cervical dislocation, 8 weeks after first inoculation, and stool, liver tissue, and cecal contents were collected. Formalin-fixed paraffin-embedded liver sections were sectioned and stained with H&E and Masson’s trichrome stain. Histology was blindly scored by a pathologist. The NAFLD Activity Score (NAS) was calculated as an unweighted sum of steatosis (0–3), ballooning (0–2), and lobular inflammation (0–3) scores. The percentage of liver tissue positive for collagen was estimated from Masson’s trichrome stains using the Visiopharm platform.

### DNA extraction, shotgun metagenomics sequencing, and bioinformatic analysis of bacterial inocula and stool

Human stool, mouse stool, and bacterial inocula were subjected to shotgun metagenomic sequencing (CosmosID Inc., Rockville, Maryland) as previously described.^18^ Bacterial abundance scores and relative abundances were used for downstream analysis of taxonomic composition, while MetaCyc pathways and enzymes (in copies per million, CPM) were used for functional annotation of the metagenome. Pathways and enzymes with CPM < 10 across all inocula and stool samples were excluded from downstream analyses.

### Mouse liver DNA extraction, 16S rRNA amplicon sequencing, and bioinformatic analysis

Microbial genomic DNA was extracted from mouse liver tissue (100–200 mg) using the DNeasy Powersoil Pro DNA kit (Cat No. 47014, QIAGEN), following homogenization. The extracted DNA samples were analyzed by 16S rRNA sequencing as previously described.^13^ Paired-end reads were de-multiplexed using QIIME. The paired-end reads were merged, followed by dereplication and length filtering using VSEARCH v2.17.1. De-noising and chimera calling were performed using the unoise3 command. Bacterial taxonomies were assigned using the SILVA database version 138. Library preparation failed for two liver samples from the GF-MCD group and two liver samples from the GF-MCD-B group.

### Bacterial culture from mouse liver samples

Liver and matching cecal content from two GF-MCD and seven GF-MCD-B representative mice were used for identification of culturable bacteria. To identify culturable bacteria from mouse liver samples, 70–100 mg of snap-frozen liver was first rinsed with 1 ml sterile anaerobic PBS by vortexing. Samples were then homogenized with 1 ml anaerobic PBS containing a 5 mm stainless steel bead in a TissueLyser LT (Qiagen) at 30 Hz for 5 min. The homogenates were plated on Columbia agar and BYESRF, comprising Brain Heart Infusion agar supplemented with 5 g of yeast extract, a mixture of mono- and disaccharides, with 500 mg of each, encompassing arabinose, fructose, fucose, galactose, mannose, ribose, xylose, cellobiose, and maltose, 5% sterilized rumen fluid (Fisher Scientific, NC1530570), essential vitamin supplements (ATCC, MD-VS), trace mineral supplements (ATCC, MD-TMS), 10 ug/ml hemin (Sigma Aldrich 51,280), 1 ug/ml vitamin K3 (Sigma), and 5 mg/ml L-Cysteine (Sigma 168,149). Snap-frozen cecal content samples were similarly processed. Colony-forming units were counted after 8 days under anaerobic conditions. The identity of each bacterial strain was confirmed by biotyping using a BRUKER Matrix-Assisted Laser Desorption/Ionization Time-Of-Flight (MALDI-TOF) mass spectrometry.

### Amino acid profiling

Amino acid profiling was performed on snap-frozen mouse stool, cecal, and liver samples by ultra-high-resolution mass spectrometry at the MD Anderson Metabolomics Core Facility. 20–30 mg of each sample was pulverized in liquid nitrogen, then homogenized with Precellys Tissue Homogenizer. A mixture of 17 stable isotope-labeled amino acids were spiked into each sample as an internal standard. Amino acids were extracted using 1 mL ice-cold 90/10 (v/v) acetonitrile/water with 0.1% formic acid. Extracts were centrifuged at 17,000*g* for 5 min at 4°C, and supernatants were transferred to clean tubes, followed by evaporation to dryness under nitrogen. Dried extracts were reconstituted in 90/10 acetonitrile/water containing 1% formic acid, then 10 μL was injected for analysis by liquid chromatography (LC)-MS. LC mobile phase A (MPA; weak) was acetonitrile containing 1% formic acid, and mobile phase B (MPB; strong) was water containing 50 mM ammonium formate. The Thermo Vanquish LC system included an Imtakt Intrada Amino Acid column (3 µm particle size, 150 × 2.1 mm) with column compartment kept at 30°C. The autosampler tray was chilled to 4°C. The mobile-phase flow rate was 300 µL/min, and the gradient elution program was as follows: 0-5 min, 15% MPB; 5–20 min, 15–30% MPB; 20–30 min, 30–95% MPB; 30–40 min, 95% MPB; 40–41 min, 95–15% MPB; 41–50 min, 15% MPB. The total run time was 50 min. Data were acquired using the Thermo Orbitrap Exploris 240 Mass Spectrometer under ESI positive ionization mode at a resolution of 240,000. Raw data files were imported to Thermo Trace Finder software for final analysis. Peaks with a signal-to-noise ratio <3 were considered undetected. The relative abundance of 37 amino acids was calculated by the individual peak area divided by total peak area.

### Statistical analysis

Differences in bacterial abundance between comparison groups were assessed using the linear discriminant analysis (LDA) effect size (LEfSe) tool^25^ with *p* < .05 and log10 LDA score >2 considered significant. Taxa that did not have ≥0.1% abundance in at least 25% of the samples were excluded. The remaining statistical analyses were performed in R (version 4.1.2; R Foundation for Statistical Computing, Vienna, Austria). Additional differential abundance analysis of taxa was performed with ANCOM v2.1,^26^ where an FDR significance threshold of 0.2 was used for calculation of W statistics. W statistics ≥ the 60^th^ percentile were considered significant. Differential abundances of MetaCyc pathways and enzymes were assessed by Mann-Whitney *U* test. Pathways/enzymes that did not have ≥0.01% abundance in at least 25% of the samples were excluded. The association between bacterial species, MetaCyc pathways/enzymes with liver fibrosis, was further determined using logistic regression. The “glm.fit” function was used to obtain odds ratios (ORs) adjusted for age and gender (AOR) and 95% confidence intervals (CIs). MetaCyc pathways/enzymes were considered to be significantly depleted if they had a Mann–Whitney *p* < .05, fold change <1, logistic regression *p* < .05, and a significant AOR indicating increased risk for the group with lower abundance and/or decreased risk for the group with higher abundance, for both liver fibrosis and advanced liver fibrosis. For *in vivo* mouse studies, statistical differences in histology scores and necropsy measurements between groups were assessed by the Mann-Whitney *U* test for ordinal histology scores, and the unpaired *t*-test for continuous variables. Statistical differences in amino acids were assessed by the Mann-Whitney *U* test. For all tests, *p* < .05 was considered to be significant.

## Results

### Microbiota changes associated with liver fibrosis in a population disproportionally affected by MASLD

**Figure 1.**
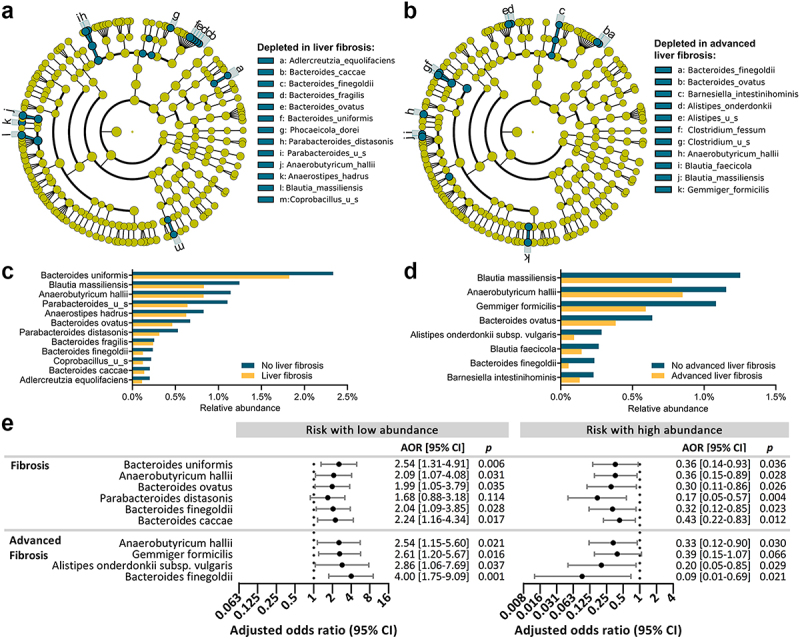
Bacterial species depleted in liver fibrosis or advanced liver fibrosis.

The study includes 340 participants from a population-based cohort disproportionally affected by MASLD and liver fibrosis.^19^ Among them, 102 (30.0%) subjects were male, 187 (55.2%) were obese and 124 (37.3%) were diabetic (Supplemental Table S1). FibroScan® VCTE was used for detection of steatosis and liver fibrosis: 229 subjects (67.6%) had liver steatosis. Forty-six subjects (13.5%) had significant liver fibrosis, while liver fibrosis was ruled out for 265 subjects (77.9%). Twenty-nine subjects (8.5%) had advanced liver fibrosis, while advanced liver fibrosis was ruled out for 303 subjects (89.1%) (Supplemental Table S1). Using stool shotgun metagenomic sequencing and LEfSe analysis, we identified 20 bacterial species that were significantly depleted (*p* < .05) in subjects with liver fibrosis and/or advanced liver fibrosis, with 13 species in subjects with liver fibrosis (Figure 1(a)) and 11 species in subjects with advanced liver fibrosis (Figure 1(b)). Among them, 12 and 8 remained significant by ANCOM analysis for liver fibrosis and advanced liver fibrosis, respectively (FDR <0.2). Species depleted in both liver fibrosis and advanced liver fibrosis included *Bacteroides finegoldii*, *Bacteroides ovatus*, and *Anaerobutyricum hallii*. Other bacterial species depleted in subjects with liver fibrosis included *Bacteroides caccae, Bacteroides uniformis*, and *Parabacteroides distasonis*, while other bacterial species depleted in subjects with advanced liver fibrosis included *Alistipes onderdonkii* and *Gemmiger formicilis* (Figure 1(c–d)). The strength of the association between these eight bacterial species and liver fibrosis or advanced liver fibrosis was further determined by logistic regression analysis (Figure 1(e)). To determine the confounding effect of common comorbidities and demographics on abundance of the 20 species associated with liver fibrosis and/or advanced liver fibrosis shown in Figure 1(a–b), redundancy analysis was performed with FibroScan liver stiffness, FibroScan CAP, diabetes, age, and gender as explanatory variables (Supplemental Figure S1). The overall model was significant (*p* = .001), but among the individual explanatory variables, only liver stiffness and age were significant (*p* = .010 and *p* = .006), while gender was borderline significant (*p* = .054). Therefore, the odds ratios calculated from logistic regression were only adjusted for age and gender. The strongest association between low bacterial abundance and advanced liver fibrosis was observed for *Bacteroides finegoldii* (AOR = 4.00 [95% CI = 1.75-9.09], *p* = .001). Remarkably, high abundance of these eight bacterial species was associated with reduced risk of liver fibrosis or advanced liver fibrosis (Figure 1(e)). The strongest protective effect was also observed for *Bacteroides finegoldii* (AOR = 0.09 [95% CI = 0.01-0.69], *p* = .021). The eight bacterial species, *Bacteroides caccae, Bacteroides ovatus, Bacteroides uniformis, Bacteroides finegoldii, Alistipes onderdonkii, Parabacteroides distasonis, Anaerobutyricum hallii*, and *Gemmiger formicilis*, were therefore selected for *in vivo* studies.

(a-b) Cladograms showing species significantly depleted (*p*<.05) in subjects with liver fibrosis (a) and advanced liver fibrosis (b). (c, d) Mean relative abundances of significant taxa in subjects with and without liver fibrosis (c) or advanced liver fibrosis (d). (e) Forest plots showing an increased risk of liver fibrosis or advanced liver fibrosis when species were at low abundance (left), and protective effect against liver fibrosis or advanced liver fibrosis when species were at high abundance (right). AORs are shown after adjusting for age and gender.

### Gut and hepatic detection of the bacterial consortium in GF mice with methionine- and choline-deficient diet-induced steatohepatitis

We first confirmed that a methionine- and choline-deficient (MCD) diet induces steatohepatitis and fibrosis in germ-free (GF) mice. GF mice were fed regular chow (GF, *n* = 7) or MCD (GF-MCD, *n* = 11) for 6 weeks. Histological assessment of livers showed a significant induction of fibrosis, steatosis, inflammation, and ceroid laden macrophages in GF-MCD mice (Supplemental Figure S2).

**Figure 2.**
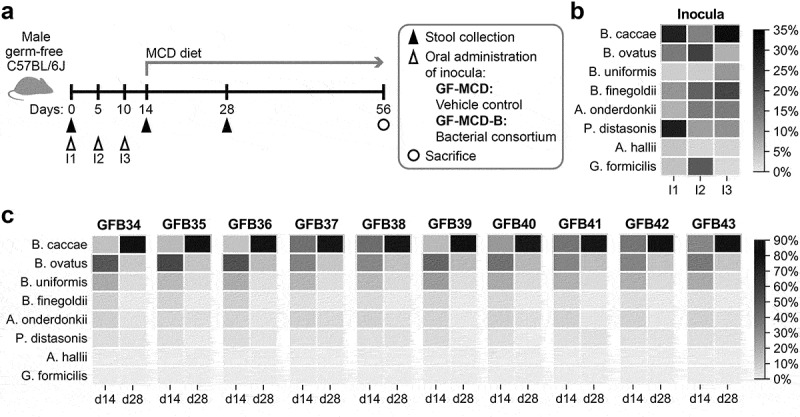
Composition of bacterial pools for inoculation and gut colonization in GF mice.

Subsequently, a consortium consisting of strains from the eight selected bacterial species (1 × 10^8^ CFU of each strain) was administered by oral gavage to GF mice (GF-MCD-B, *n* = 10) on days 0, 5, and 10 (inocula I1, I2, I3, Figure 2(a)). Control mice received oral gavage of PBS (GF-MCD, *n* = 11). On day 14, mice in both groups were switched from regular chow to MCD for 6 additional weeks to induce steatosis and liver fibrosis. Shotgun metagenomic sequencing of the three inocula confirmed the presence of all eight bacterial strains and lack of contamination from other strains (Figure 2(b)). However, relative abundances of the eight bacterial strains in the three inocula differed with an overall high abundance of *Bacteroides caccae* (mean = 24.5%, range = 12.9-32.5%), *Parabacteroides distasonis* (mean = 16.3% range = 9.2-28.8%), *Bacteroides finegoldii* (mean = 16.4%, range = 10.3-21.7%), *Bacteroides ovatus* (mean = 14.3%, range = 6.8-22.5%), and *Alistipes onderdonkii* (mean = 11.4%, range = 6.7-13.9%), while abundance was low for *Bacteroides uniformis* (mean = 5.6%, range = 3.3-10.0%), *Anaerobutyricum hallii* (mean = 3.0%, range = 2.3-4.3%), and *Gemmiger formicilis* (mean = 8.4%, range = 2.0-18.5%).

(a) Schematic study design to determine the effect of bacterial inoculation on liver fibrosis prevention *in vivo*. (b) Relative abundances of the eight bacterial strains in inoculum used to treat mice on days 0 (I1), 5 (I2), and 10 (I3). (c) Relative abundances of the eight bacterial strains in the stool of GF-MCD-B mice collected at days 14 and 28 after three inoculations. Total absence of bacterial strains was confirmed at d0, prior to any inoculation (not shown). For each mouse, heatmaps show the relative abundance of each bacterial strain over time.

Shotgun metagenomic sequencing of stool pellets confirmed the absence of bacteria in GF-MCD on days 0 and 28, and in GF-MCD-B mice on day 0 prior to inoculation, suggesting lack of any contamination. On days 14 and 28, six of the eight bacterial species successfully colonized the gut of the mice in the GF-MCD-B group (*Bacteroides finegoldii, Bacteroides caccae, Bacteroides ovatus, Bacteroides uniformis, Parabacteroides distasonis, Alistipes onderdonkii*) (Figure 2(c)). These six strains were detected in all GF-MCD-B mice at both 14 and 28 days, except for *Parabacteroides distasonis*, which was detected in 8 of 10 mice on day 28. Overall, stool taxonomic composition was very similar across mice. Despite not being the most abundant strain in the inocula, *Bacteroides ovatus* was the most abundant strain (31.2%) at 14 days. At 28 days, *Bacteroides caccae* became the most abundant strain (77.3%).

**Figure 3.**
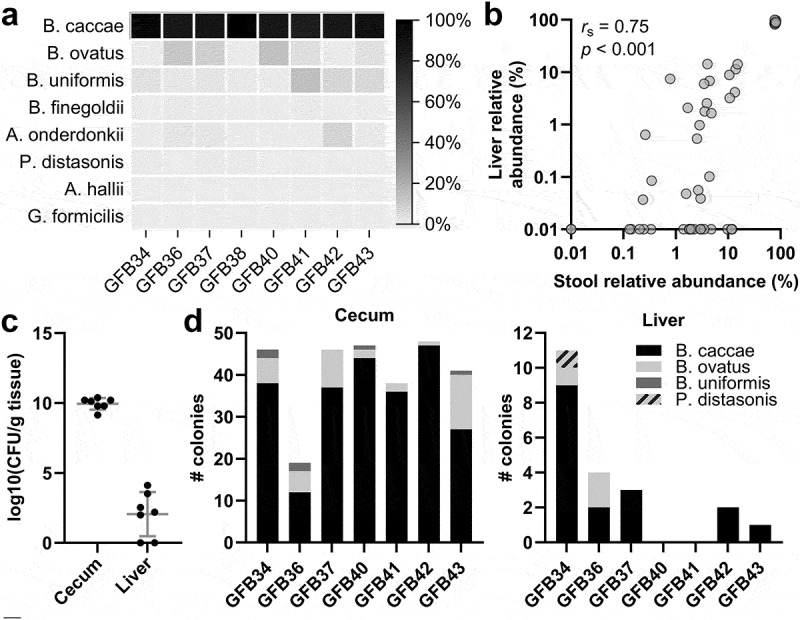
Detection of an intrahepatic microbiome after inoculation of GF mice.

None of the eight species were detected in the livers of GF-MCD mice. In contrast, all six species that successfully colonized the gut were detected in liver samples of GF-MCD-B mice. Similar to stool at 28 days, *Bacteroides caccae* was the most abundant species in the liver, with presence detected in all eight GF-MCD-B mice and a median relative abundance of 86.5% (Figure 3(a)), followed by *Bacteroides uniformis* (detected in the livers of six GF-MCD-B mice, median abundance of 2.2%). The most abundant species in stool were also the most abundant species in the liver at day 28 (*r*_s_ = 0.75, *p* < .001) (Figure 3(b)). The presence of live bacteria in the liver was confirmed by bacterial culture. While no colony-forming units were detected in liver from two control GF-MCD mice, colonies were detected in five out of seven livers of GF-MCD-B mice (Figure 3(c)). As anticipated, the number of colony-forming units was considerably lower in the liver compared to the cecal content of the same mice (Figure 3(c)). The culturable bacteria were predominantly *Bacteroides caccae* in both cecal and liver samples (Figure 3(d)).

(a) Relative abundance of the eight bacterial strains in the liver of GF-MCD-B mice at time of necropsy, as determined by 16S rRNA sequencing. (b) Spearman’s correlation between relative abundances of the bacterial species in the liver and in stool collected at day 28. (c) Levels of culturable bacteria in the cecal content and liver of GF-MCD-B mice. No culturable bacteria were detected in GF-MCD mice. Error bars show mean with SD. (d) Distribution of bacterial species cultured from each GF-MCD-B liver, as determined by biotyping. Up to 24 colonies were biotyped per sample.

### Prevention of liver fibrosis by the bacterial consortium in mcd-treated mice

**Figure 4.**
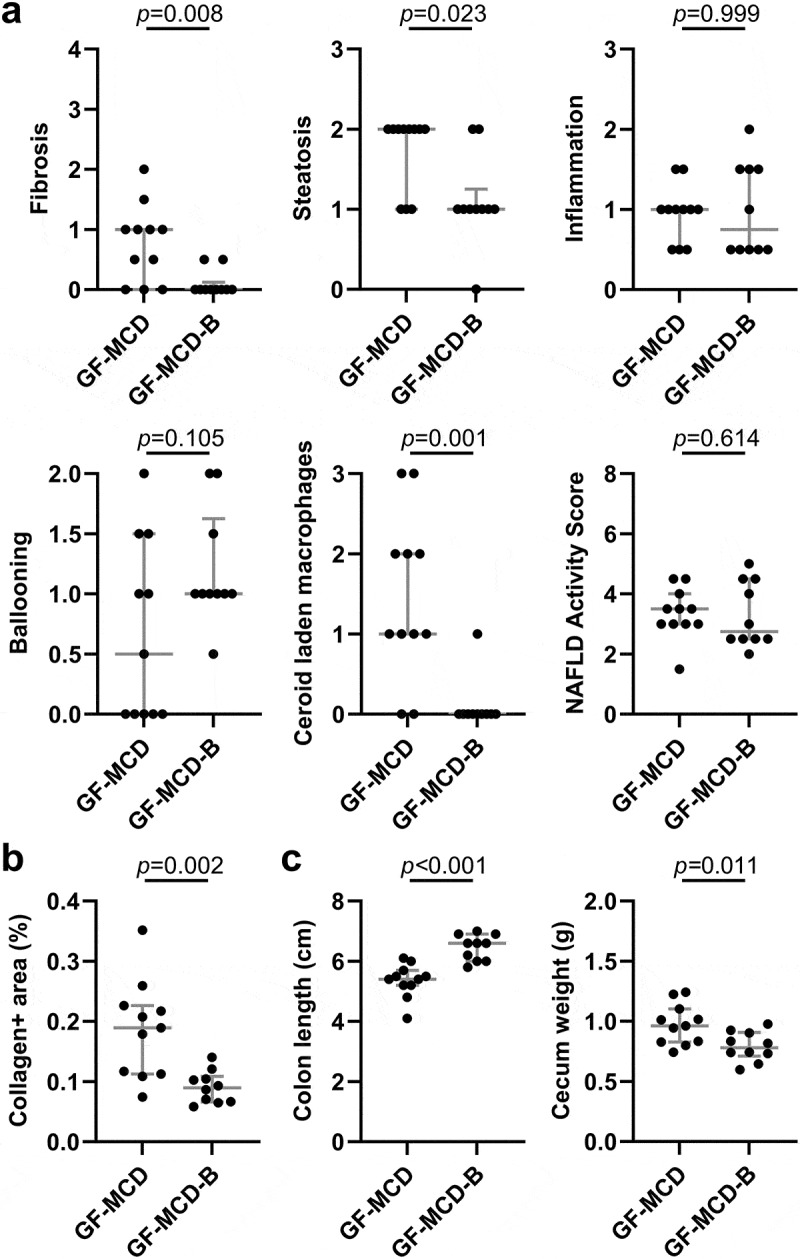
Prevention of mcd-induced liver fibrosis by the selected bacterial strains in GF mice.

At time of necropsy, body weight and spleen weight were significantly greater in GF-MCD-B mice. However, no significant differences in the liver-to-body weight ratio and spleen-to-body weight ratio were observed (Supplemental Figure S3). Histological assessment of livers at necropsy showed that while there was no significant change in inflammation, ballooning, or NAFLD Activity Scores between GF-MCD and GF-MCD-B mice, the bacterial consortium significantly reduced liver fibrosis (median score of 0 in GF-MCD-B mice vs 1 in GF-MCD mice, *p* = .008), liver steatosis (1 versus 2, *p* = .023), and ceroid laden macrophages (0 versus 1, *p* = .001) (Figure 4(a)). Notably, liver fibrosis was detected in only 2/10 (20%) GF-MCD-B mice, versus 8/11 (73%) GF-MCD mice. The percentage of collagen-positive areas in the liver also significantly decreased in GF-MCD-B mice (Figure 4(b)). Additionally, GF-MCD-B mice had significantly increased colon length (6.6 vs 5.4 cm, *p* < .001) and decreased cecum weight (0.78 g vs 0.96 g, *p* = .011) (Figure 4(c)).

(a-c) Liver histological assessment of H&E stains (a), hepatic quantification of collagen-positive areas in Masson’s trichrome stains (b) and necropsy measurements (c) of GF mice under MCD treatment for 6 weeks, without bacterial inoculation (GF-MCD, *n* = 11) or inoculated with the pool of eight bacterial strains (GF-MCD-B, *n* = 10). Error bars show median with interquartile range. Significance was assessed by the Mann-Whitney *U* test for histological scores, and unpaired *t*-test for collagen-positive areas and necropsy measurements.

### Functional mechanisms correlating with anti-fibrotic effect of the inoculated bacterial consortium: a human-to-mouse comparative metagenomic functional analysis

**Figure 5.**
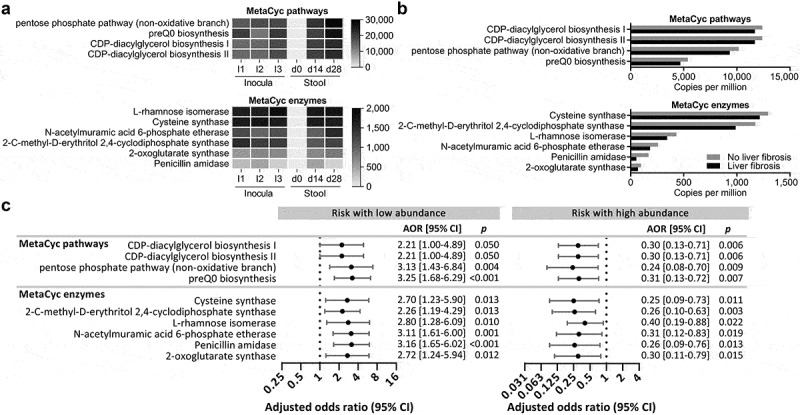
Metagenomic functions negatively associated with liver fibrosis in the cohort subjects and enriched in GF-MCD-B mice after bacterial inoculation.

To identify mechanisms by which the selected bacterial species may exert anti-fibrotic effects, we performed a comparative analysis between the metagenomic functional profiles of human and mouse samples. We first characterized the stool metagenomic functions negatively associated with liver fibrosis in the human cohort. Eight MetaCyc pathways and 26 MetaCyc enzymes were significantly depleted in subjects with liver fibrosis and advanced liver fibrosis. Their low abundance was significantly associated with increased risk for liver fibrosis or advanced liver fibrosis, while their high abundance was significantly associated with protection against liver fibrosis or advanced liver fibrosis (Supplemental Table S3). We then characterized the stool metagenomic functions enriched in GF-MCD-B mice after bacterial inoculation. Ninety-four MetaCyc pathways and 328 MetaCyc enzymes were detected in all three bacterial inocula and also enriched in all 10 GF-MCD-B mice at days 14 and 28 after first inoculation (Supplemental Table S4). Integrating both human and mouse datasets, four pathways and six enzymes were enriched in the stool of mice after bacterial inoculation (Figure 5(a)), significantly depleted in the cohort subjects with liver fibrosis (Figure 5(b)), significantly associated with liver fibrosis when detected at low abundance in the cohort subjects, and significantly associated with protection against liver fibrosis when detected at high abundance in the cohort subjects (Figure 5(c)). The four MetaCyc pathways included pentose phosphate pathway (non-oxidative branch) I (AOR(low) = 3.13 [95% CI = 1.43-6.84], *p* = .004; AOR(high) = 0.24 [95% CI = 0.08-0.70], *p=*.009) and preQ0 biosynthesis (AOR(low) = 3.25 [95% CI = 1.68-6.29], *p* ≤ .001; AOR(high) = 0.31 [95% CI = 0.13-0.72], *p* = .007). The six enzymes included penicillin amidase (AOR(low) = 3.16 [95% CI = 1.65-6.02], *p* ≤ .001; AOR(high) = 0.26 [95% CI = 0.09-0.76], *p* = .013) and cysteine synthase (AOR(low) = 2.70 [95% CI = 1.23-5.90], *p* = .013; AOR(high) = 0.25 [95% CI = 0.09-0.73], *p* = .011) (Figure 5(c)).

(a) MetaCyc pathways and enzymes negatively associated with both fibrosis and advanced fibrosis in the 340 cohort subjects, and enriched in the gut microbiota of GF-MCD-B after bacterial inoculation. Abundance (CPM) of these pathways and enzymes is shown for the inocula used to treat mice on days 0 (I1), 5 (I2), and 10 (I3), and median abundances in the stool of GF-MCD-B mice before (d0) and after inoculations (d14, d28). (b) Abundances of MetaCyc pathways and enzymes significantly depleted in the gut metagenome of the cohort subjects with liver fibrosis. (c) Forest plot showing the increased risk of liver fibrosis in cohort subjects when the stool abundance of the identified MetaCyc pathways and enzymes were at low abundance (left), and the protective effect against liver fibrosis in cohort subjects when the stool abundance of MetaCyc pathways and enzymes were present at high abundance (right).

### Contribution of amino acids to the anti-fibrotic effect of the bacterial consortium

**Figure 6.**
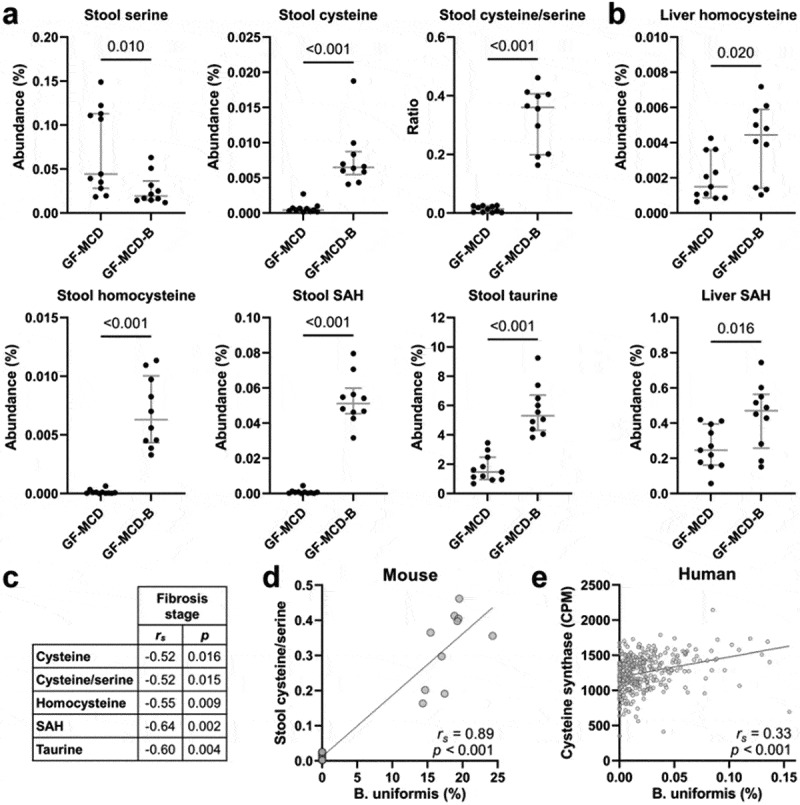
Altered abundances of cysteine-related amino acids in GF-MCD-B mice after bacterial inoculation.

The integrated functional metagenome analysis identified cysteine synthase as a potential mediator of liver fibrosis protection in humans. This enzyme is part of a *de novo* pathway of cysteine synthesis, that is absent in mammals and involves a two-step conversion of L-serine to L-cysteine. To determine whether the bacterial inoculations altered host levels of cysteine, serine, and other related amino acids, comprehensive profiling of 37 amino acids was performed on stool, cecum, and liver tissue collected from GF-MCD and GF-MCD-B mice at necropsy. The largest differences in abundance in GF-MCD-B mice, among all 37 amino acids measured, were increases in relative cysteine levels (FC = 14.93, *p* < .001) and cysteine-to-serine ratios (FC = 25.92, *p* < .001), indicating a strong activation of cysteine synthase (Supplemental Table S5; Figure 6(a)). Large increases were also observed in stool levels of the downstream amino acids of this pathway such as homocysteine (FC = 93.80, *p* < .001), S-adenosylhomocysteine (SAH) (FC = 75.73, *p* < .001) and taurine (FC = 3.63, *p* < .001) (Figure 6(a)). Similarly, strong increases in levels of cysteine (FC = 11.58, *p* < .001), cysteine-to-serine ratio (FC = 38.85, *p* < .001), homocysteine (FC = 76.57, *p* < .001), SAH (FC = 26.85, *p* < 0.001), and taurine (FC = 2.65, *p* < .001) were also observed in the cecum (Supplemental Table S5). Finally, increased hepatic levels of homocysteine (FC = 2.96, *p* = .020) and SAH (FC = 1.91, *p* = .016) were also found in GF-MCD-B mice (Figure 6(b)). Importantly, strong negative correlations were observed between fibrosis scores and stool cysteine-to-serine ratio (*r*_*s*_ = −0.52, *p* = .015), cysteine (*r_s_* = −0.52, *p* = .016), homocysteine (*r_s_* = −0.55, *p* = .009), SAH (*r*_*s*_ = −0.64, *p* = .002), and taurine (*r_s_* = −0.60, *p* = .004) (Figure 6(c)). In the cecum, similar significant negative correlations with fibrosis scores were observed (cysteine-to-serine ratio: *r_s_* = −0.48, *p* = .028; cysteine: *r_s_* = −0.46, *p* = .036; homocysteine: *r_s_* = −0.53; *p* = .014; SAH: *r_s_* = −0.58, *p* = .006; taurine: *r_s_* = −0.61, *p* = .003). While all six of the colonizing strains possess the cysteine synthase enzyme, only *Bacteroides uniformis* ATCC 8492 also possesses serine acetyltransferase, the first enzyme of the two-step *de novo* cysteine synthesis pathway (Supplemental Table S6). The role of *Bacteroides uniformis* in the observed activation of the cysteine synthesis pathway was further suggested by a strong correlation between *Bacteroides uniformis* abundance and cysteine-to-serine ratios in stool (Figure 6(d), *r*_*s*_ = 0.89, *p* < .001), while none of the other bacterial species showed a significant correlation. In the human cohort, stool *Bacteroides uniformis* abundance was also correlated with abundance of cysteine synthase (Figure 6(e)), further suggesting a contribution of *Bacteroides uniformis* to cysteine synthase activity in the gut.

(a, b) Stool levels of cysteine-related amino acids (a) and hepatic levels of homocysteine and SAH (b), in mice without bacterial inoculation (GF-MCD, *n* = 11) or inoculated with the bacterial consortium (GF-MCD-B, *n* = 10). Error bars show median with interquartile range. Significance was assessed by the Mann-Whitney *U* test. (c) Spearman’s correlation coefficients and *p*-values between liver fibrosis score and stool levels of cysteine-related amino acids. (d) Spearman’s correlation between abundance of *Bacteroides uniformis* and cysteine-to-serine ratios in mice stool. (e) Spearman’s correlation between abundance of *Bacteroides uniformis* and the cysteine synthase gene in stool from the human cohort.

**Figure 7.**
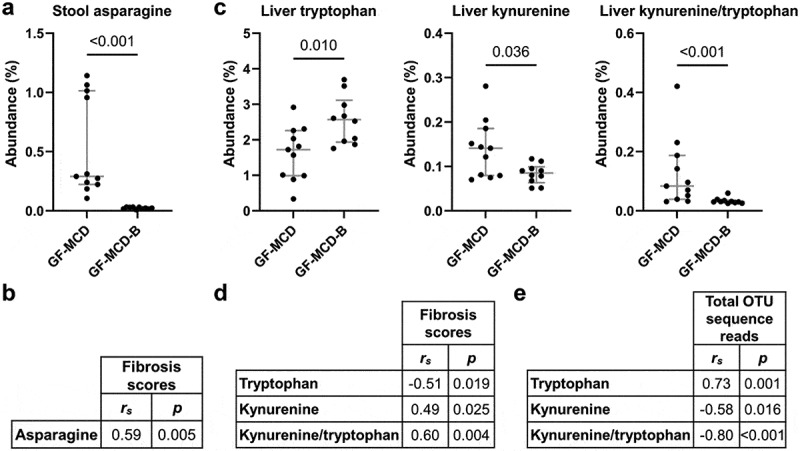
Altered metabolism of asparagine and tryptophan in GF-MCD-B mice after bacterial inoculation.

The other major change in amino acid abundance was a sharp decrease in asparagine levels in the stool (FC = −12.59, *p* < .001, Figure 7(a)) and cecum (FC = −44.55, *p* < .001) of GF-MCD-B mice. Liver fibrosis scores were significantly correlated with asparagine levels in both the stool (*r*_*s*_ = 0.59, *p* = .005) and cecum (*r_s_* = 0.61, *p* = .003) (Figure 7(b)). All six colonizing species possess the asparaginase enzyme responsible for L-asparagine degradation (Supplemental Table S6).

(a) Stool levels of asparagine in mice without bacterial inoculation (GF-MCD, *n* = 11) or inoculated with the bacterial consortium (GF-MCD-B, *n* = 10). Error bars show median with interquartile range. Significance was assessed by the Mann-Whitney *U* test. (b) Spearman’s correlation between stool levels of asparagine and liver fibrosis scores. (c) Hepatic levels of tryptophan, kynurenine, and tryptophan-to-kynurenine ratio in GF-MCD and GF-MCD-B mice. Error bars show median with interquartile range. Significance was assessed by the Mann-Whitney *U* test. (d) Spearman’s correlation between hepatic levels of tryptophan, kynurenine, and the tryptophan-to-kynurenine ratio and liver fibrosis scores. (e) Spearman’s correlation between hepatic levels of tryptophan, kynurenine, and the tryptophan-to-kynurenine ratio, and total OTU sequence reads in the liver.

Finally, an increase in tryptophan (FC = 1.49, *p* = .010) and decrease in its product kynurenine (FC = −1.66, *p* = .036) and kynurenine-to-tryptophan ratio (FC = −2.73, *p* = .001) was observed in the livers of GF-MCD-B mice, but not in the stool nor cecum (Figure 7(c)). Hepatic levels of kynurenine-to-tryptophan ratios are positively correlated with liver fibrosis scores (*r*_*s*_ = 0.60, *p* = .004) (Figure 7(d)) and negatively correlated with total hepatic OTU sequence reads (*r_s_* = −0.80, *p* < .001) (Figure 7(e)).

## Discussion

In this study, we first identified bacterial species with a negative association with liver fibrosis in a high-risk population cohort. We subsequently tested a bacterial consortium for its potential protective effect against liver fibrosis, in a diet-induced mouse model of MASLD. In addition, we identified gut metagenomic functions enriched in mice following inoculation with the consortium, and validated the findings by amino acid profiling. We first observed an association between increased risk of liver fibrosis and low abundance of *Bacteroides* species (*Bacteroides caccae, Bacteroides finegoldii, Bacteroides ovatus*, *Bacteroides uniformis*) and *Parabacteroides distasonis*, in a population cohort disproportionally affected by MASLD and liver fibrosis. Additional discoveries included the association between low abundance of *Alistipes onderdonkii* subsp. *Vulgaris*, *Anaerobutyricum hallii*, and *Gemmiger formicilis* with increased risk of liver fibrosis. Depletion of *Parabacteroides*, *Bacteroides*, and *Alistipes* species has been previously associated with steatohepatitis and liver fibrosis severity in other human studies.^27–29^ In mice, *Parabacteroides distasonis* administration improved features of MASLD and liver fibrosis.^30,31^

Subsequently, we tested the ability of eight bacterial strains selected from human cohort data, to prevent MCD-induced liver fibrosis in GF mice. Only six strains successfully colonized the gut of these mice. Lack of successful colonization by *Anaerobutyricum hallii* and *Gemmiger formicilis* may be due to loss of viability during preparation of the inocula, or lower competitiveness compared with the other bacterial strains in the consortium. Temporal variation in gut microbiota composition in the first 1–2 weeks after inoculation in GF mice has been reported.^23^ Six colonizing strains in the gut could also be detected in liver tissues. Strains with higher abundance in the stool also had higher abundance in the liver. The presence of an intrahepatic microbiota has recently been reported. While bacterial DNA has been detected in the liver of healthy individuals, total amounts are increased in obesity and MASH, correlating with fatty liver index and histology severity.^32,33^ We used culturing of live bacteria from liver tissues as a complementary assay to demonstrate the presence of live colonizing bacteria in the liver. While the inoculated bacteria detected in liver are likely originating from the portal circulation, it remains to be determined whether their presence is transient, or they would become resident intrahepatic populations.^34^

Pre-treatment of GF mice with the bacterial consortium significantly reduced liver steatosis, liver fibrosis, and the presence of ceroid laden macrophages in the liver, a marker of liver injury and chronic oxidative stress.^35^ It also altered morphologic features of the gut, including increased colon length and decreased cecal weight. Shortened colon length is a marker of colonic inflammation.^36^ The reduction in cecal weight reflects the abrogation of the enlarged cecum, which is a hallmark of GF mice.^37^ The increased colon length suggests a possible improvement in colonic inflammatory status.

Cross-talk between gut microbiota, host/bacterial metabolism, and immunity has been implicated in MASLD pathology.^38,39^ By comparing the pathways/enzymes associated both with the presence of the bacterial consortium and with liver fibrosis in the human cohort, we observed an enrichment of cysteine synthase, which is part of a *de novo* pathway of cysteine synthesis involving a two-step conversion of L-serine to L-cysteine, absent in mammalian cells. Instead, mammals generate cysteine by converting homocysteine to cysteine. Enrichment of cysteine synthase activity in the stool and cecum upon bacterial inoculation was indeed accompanied by increased conversion of serine to cysteine as assessed by amino acid profiling. We also observed increased stool and cecal levels of homocysteine, SAH, and taurine. Most importantly, stool levels of cysteine, cysteine/serine ratio, homocysteine, SAH, and taurine were all negatively correlated with fibrosis scores in the mice. Taurine is a major downstream product of cysteine oxidation, which protects against features of MASLD *in vivo*, including inflammation, steatosis, fibrosis, and oxidative stress.^40^ *In vivo* studies have suggested the role of N-acetylcysteine (NAC), an acetylated precursor to cysteine, in preventing hepatic lipid accumulation, oxidative stress, and inflammation in MASLD.^41^ Among all bacteria in the consortium, increased activity of the *de novo* pathway of cysteine synthesis, was likely dependent upon the presence of *Bacteroides uniformis*, the only strain to possess both genes in this pathway. Confirming this hypothesis, strong correlations were observed between *Bacteroides uniformis* abundance and cysteine-to-serine ratios in mouse stool, and between *Bacteroides uniformis* and the cysteine synthase enzyme in human stool. While the protective effect of *Bacteroides uniformis* against steatosis, liver injury, and inflammation, has been previously reported in mice,^42–44^ this is the first study demonstrating a protective role against liver fibrosis and implicating bacterial cysteine synthase function. The possible effects of cysteine supplementation or cysteine synthase inhibition on liver fibrosis should be further investigated. However, cysteine solutions have notably poor resistance against oxidation and precipitation, as well as toxicity.^45^ When administered orally, the cysteine prodrug NAC is rapidly absorbed in the intestines and metabolized by the liver. It is unknown whether cysteine levels in the gut are altered after NAC administration, which is the phenotype we observed with bacterial pool administration. Furthermore, targeting cysteine synthase activity with inhibitors could have unintended effects on gut microbiota composition and activity.^46^

A strong depletion of asparagine in stool and cecum, correlating with reduced liver fibrosis severity, was also observed following bacterial treatment. Asparagine is sourced from the diet or synthesized by asparagine synthetase and metabolized by asparaginase. All six colonizing species were predicted to possess asparaginase activity, which hydrolyzes asparagine into aspartic acid and ammonia. Gut microbial communities with high *Bacteroides* abundance have been associated with increased asparaginase activity.^47^ Finally, we observed a decreased conversion of tryptophan to kynurenine specifically in the liver, which correlated with reduced liver fibrosis severity. Interestingly, tryptophan levels are positively correlated with hepatic bacterial load, suggesting that bacteria presence in the liver mediated the loss of conversion to kynurenine. Quantification of selected amino acids in stool or blood of MASLD patients may provide a noninvasive method of monitoring liver fibrosis progression and identifying patients at high risk who would benefit from preventive therapeutic strategies involving probiotics and/or amino acid supplementation.

One limitation of this study is that there is no ideal preclinical model demonstrating all histological and metabolic features of human MASLD.^48^ In addition, this study utilized germ-free mice without any preexisting gut microbiota, which does not address the colonization ability in conventionalized hosts, thus limiting the generalizability of the findings.

In conclusion, we identified taxonomic changes associated with reduced risk of liver fibrosis in a high-risk population. Based on these findings, we tested the ability of a bacterial consortium of eight species to prevent MASLD-related liver fibrosis *in vivo*. Treatment with the consortium conferred protection against liver fibrosis in a diet-induced, GF mouse model of steatohepatitis. Presence of the bacterial species was detected in both the stool and liver. Comparative analysis of metagenomic functional profiles in humans and mice identified pathways and enzymes, likely to mediate the protective effect against liver fibrosis and of relevance to human disease. The role of bacterial cysteine metabolism was further suggested by amino acid profiling. Other liver fibrosis-associated amino acid pathways included asparaginase activity in stool and tryptophan-to-kynurenine conversion in the liver. Amino acid profiling in human stool samples would be beneficial in confirming the clinical relevance of these amino acid associations. These findings support further development of live biotherapeutic products for the prevention of liver fibrosis in MASLD and provide novel insights into their mechanism of action.

## Supplementary Material

Supplemental Material

## Data Availability

The shotgun metagenomic sequencing data of human stool has been deposited into the Sequence Read Archive (SRA) of the National Center for Biotechnology Information (NCBI) under BioProject accession number PRJNA734860 (https://www.ncbi.nlm.nih.gov/bioproject/PRJNA734860).
