# Supplementary material for: Protection against fibrosis by a bacterial consortium in metabolic dysfunction-associated steatohepatitis and the role of amino acid metabolism
Source: Gut Microbes. 2024 Sep 6;16(1):2399260. doi: 10.1080/19490976.2024.2399260 (PMC11382720; doi:10.1080/19490976.2024.2399260)
Supplement: Supplemental Material [file KGMI_A_2399260_SM1162.zip › Supplementary_Tables_Figures.docx]

**Supplementary Tables and Figures**

**Supplement to:** **S.Y. Kwan, K.A. Gonzales, M.A. Jamal et al. Protection against fibrosis by a bacterial consortium in metabolic dysfunction-associated steatohepatitis and the role of amino acid metabolism**

Table of Contents

[SUPPLEMENTARY TABLES 2](#_Toc172909308)

[Table S1. 2](#_Toc172909309)

[Table S2. 3](#_Toc172909310)

[Table S3. 7](#_Toc172909311)

[Table S4. 9](#_Toc172909312)

[Table S5. 16](#_Toc172909313)

[Table S6. 17](#_Toc172909314)

[SUPPLEMENTARY FIGURES 18](#_Toc172909315)

[Figure S1. 18](#_Toc172909316)

[Figure S2. 19](#_Toc172909317)

[Figure S3. 20](#_Toc172909318)

# SUPPLEMENTARY TABLES

Table S1. **Demographic and clinical parameters of the 340 study participants.** Data are presented as frequency (%) for categorical variables, or mean (range) – median for continuous variables. BMI, body mass index; HbA1c, hemoglobin A1c; AST, aspartate aminotransferase; ALT, alanine aminotransferase; HDL, high-density lipoprotein; LDL, low-density lipoprotein.

| **Parameters** | |  |
| --- | --- | --- |
| **Male (n=340)** | | 102 (30.0%) |
| **Age (n=340)** | | 55.1 (18.0-89.0) - 57.0 |
| **BMI (n=339)** | | 31.4 (16.7-50.0) - 30.8 |
| **Obese (n=339)** | | 187 (55.2%) |
| **Diabetes (n=332)** | | 124 (37.3%) |
| **HbA1c (%) (n=338)** | | 6.6 (4.8-16.0) - 6.0 |
| **Waist circumference (cm) (n=340)** | | 104.1 (71.0-143.0) - 104.0 |
| **Waist-to-hip ratio (n=322)** | | 0.9 (0.7-1.1) - 0.9 |
| **Hypertension (n=340)** | | 118 (34.7%) |
| **FibroScan CAP (dB/m) (n=339)** | | 290.7 (100.0-400.0) - 298.0 |
| **Liver steatosis (CAP≥268) (n=339)** | | 229 (67.6%) |
| **FibroScan LSM (kPa) (n=340)** | | 5.7 (1.9-45.5) - 4.6 |
| **No liver fibrosis (LSM<6.2 kPa)** | | 265 (77.9%) |
| **Fibrosis (LSM≥7.3 kPa)** | | 46 (13.5%) |
| **No advanced fibrosis (LSM<8.0 kPa)** | | 303 (89.1%) |
| **Advanced fibrosis (LSM≥8.8 kPa) (n=340)** | | 29 (8.5%) |
| **NAFLD score (n=331)** | | -1.2 (-5.5-8.9) - -1.1 |
| **APRI (n=337)** | | 0.3 (0.1-2.3) - 0.2 |
| **Alcohol intake (g/day) (n=323)** | | 3.4 (0.0-325.0) - 0.0 |
| **Drinking status (n=323)** | |  |
|  | Never | 213 (65.9%) |
|  | Moderate | 94 (29.1%) |
|  | Heavy | 16 (5.0%) |
| **Smoking status (n=323)** | |  |
|  | Never | 233 (72.1%) |
|  | Former | 67 (20.7%) |
|  | Current | 23 (7.1%) |
| **Blood tests** | |  |
|  | AST (U/L) (n=337) | 21.7 (6.0-205.0) - 19.0 |
|  | Abnormal AST (n=337) | 29 (8.6%) |
|  | ALT (U/L) (n=337) | 32.4 (12.0-173.0) - 27.0 |
|  | Abnormal ALT (n=337) | 114 (33.8%) |
|  | Total bilirubin (mg/dL) (n=338) | 0.5 (0.1-1.9) - 0.5 |
|  | Creatinine (mg/dL) (n=338) | 0.8 (0.4-5.0) - 0.7 |
|  | Albumin (mg/dL) (n=338) | 3.9 (3.0-4.6) - 3.9 |
|  | Alkaline phosphatase (U/L) (n=338) | 90.1 (38.0-165.0) - 86.0 |
|  | Fasting glucose (mg/dL) (n=323) | 114.0 (70.0-360.0) - 97.0 |
|  | Triglycerides (mg/dL) (n=338) | 153.0 (33.0-1596.0) - 126.0 |
|  | Total cholesterol (mg/dL) (n=337) | 184.8 (50.0-318.0) - 185.0 |
|  | HDL cholesterol (mg/dL) (n=338) | 51.6 (0.0-109.0) - 49.5 |
|  | LDL cholesterol (mg/dL) (n=332) | 104.0 (8.0-204.0) - 105.0 |
|  | Platelets (x10^9^/L) (n=338) | 251.2 (116.0-480.0) - 247.0 |

Table S2. **Ingredient lists and chemical compositions of the regular chow (Select Rodent 50 IF/6F Auto Diet, #50VF, LabDiet) and of the purified methionine- and choline-deficient diet (#A02082002BR, Research Diets, double irradiated, with 1.5X vitamins).**

**Regular chow**

| **INGREDIENTS** | |  |  |
| --- | --- | --- | --- |
|  | Ground Wheat, Ground Corn, Corn Gluten Meal, Wheat Middlings, Soybean Oil, Calcium Carbonate, Dicalcium Phosphate, Brewers Dried Yeast, L-Lysine, Menadione Dimethylpyrimidinol Bisulfite (Vitamin K), Salt, Potassium Chloride, Chromium Potassium Sulfate, Pyridoxine Hydrochloride, DL-Methionine, Choline Chloride, Thiamine Mononitrate, L-Tryptophan, Casein, Magnesium Oxide, Calcium Pantothenate, Vitamin A Acetate, Folic Acid, Cholecalciferol (Vitamin D3), DL-Alpha Tocopheryl Acetate (Vitamin E), Vitamin B12 Supplement, Riboflavin Supplement, Manganous Oxide, Preserved with Mixed Tocopherols, Zinc Oxide, Ferrous Carbonate, Citric Acid (a Preservative), Nicotinic Acid, Copper Sulfate, Ferrous Sulfate, Zinc Sulfate, Calcium Iodate, Rosemary Extract, Biotin, Cobalt Carbonate, Sodium Selenite. | | |
| **CHEMICAL COMPOSITION** | |  |  |
| **Protein** |  |  |  |
|  | Arginine | 0.7 | % |
|  | Cystine | 0.37 | % |
|  | Glycine | 0.58 | % |
|  | Histidine | 0.39 | % |
|  | Isoleucine | 0.76 | % |
|  | Leucine | 2.33 | % |
|  | Lysine | 1.16 | % |
|  | Methionine | 0.61 | % |
|  | Phenylalanine | 1.01 | % |
|  | Tyrosine | 0.71 | % |
|  | Threonine | 0.6 | % |
|  | Tryptophan | 0.26 | % |
|  | Valine | 0.85 | % |
|  | Serine | 0.99 | % |
|  | Aspartic Acid | 1.36 | % |
|  | Glutamic Acid | 4.79 | % |
|  | Alanine | 1.44 | % |
|  | Proline | 1.93 | % |
|  | Taurine | 0 | % |
| **Fat** |  |  |  |
|  | Fat (ether extract) | 6.1 | % |
|  | Fat (acid hydrolysis) | 7.7 | % |
|  | Cholesterol | 0 | ppm |
|  | Linoleic Acid | 3.32 | % |
|  | Linolenic Acid | 0.41 | % |
|  | Arachidonic Acid | 0.01 | % |
|  | Omega-3 Fatty Acids | 0.38 | % |
|  | Total Saturated Fatty Acids | 1 | % |
|  | Total Monounsaturated Fatty Acids | 1.49 | % |
| **Fiber** |  |  |  |
|  | Fiber (Crude) | 2.4 | % |
|  | Neutral Detergent Fiber | 12.3 | % |
|  | Acid Detergent Fiber | 3.7 | % |
| **Carbohydrates** | |  |  |
|  | Nitrogen-Free Extract (by difference) | 57.5 | % |
|  | Starch | 43.3 | % |
|  | Sucrose | 0.32 | % |
|  | Total Digestible Nutrients | 79.3 | % |
|  | Gross Energy | 4.32 | kcal/g |
|  | Physiological Fuel Value | 3.61 | kcal/g |
|  | Metabolizable Energy | 3.41 | kcal/g |
| **Minerals** |  |  |  |
|  | **Ash** | 0.05 | % |
|  | Calcium | 1.05 | % |
|  | Phosphorus | 0.55 | % |
|  | Phosphorus (non-phytate) | 0.34 | % |
|  | Potassium | 0.52 | % |
|  | Magnesium | 0.18 | % |
|  | Sulfur | 0.21 | % |
|  | Sodium | 0.22 | % |
|  | Chloride | 0.75 | % |
|  | Fluorine | 23 | ppm |
|  | Iron | 240 | ppm |
|  | Zinc | 110 | ppm |
|  | Manganese | 140 | ppm |
|  | Copper | 15 | ppm |
|  | Cobalt | 0.47 | ppm |
|  | Iodine | 1.5 | ppm |
|  | Chromium (added) | 0.97 | ppm |
|  | Selenium | 0.33 | ppm |
| **Vitamins** |  |  |  |
|  | Carotene | 2.5 | ppm |
|  | Vitamin K | 22 | ppm |
|  | Thiamin | 124 | ppm |
|  | Riboflavin | 27 | ppm |
|  | Niacin | 120 | ppm |
|  | Pantothenic Acid | 140 | ppm |
|  | Choline | 2000 | ppm |
|  | Folic Acid | 8.6 | ppm |
|  | Pyridoxine | 26 | ppm |
|  | Biotin | 0.9 | ppm |
|  | B12 | 130 | mg/kg |
|  | Vitamin A | 30 | IU/g |
|  | Vitamin D3 (added) | 2.3 | IU/g |
|  | Vitamin E | 140 | IU/kg |
|  | Ascorbic Acid | 0 | mg/g |

**Methionine- and choline-deficient diet**

| **Protein** | |  |  |  |
| --- | --- | --- | --- | --- |
|  |  | L-Alanine | 3.5 | g |
|  |  | L-Arginine | 12.1 | g |
|  |  | L-Asparagine-H2O | 6.0 | g |
|  |  | L-Aspartate (aka aspartic acid) | 3.5 | g |
|  |  | L-Cystine | 3.5 | g |
|  |  | L-Glutamine | 40.0 | g |
|  |  | Glycine | 23.3 | g |
|  |  | L-Histidine-HCl-H2O | 4.5 | g |
|  |  | L-Isoleucine | 8.2 | g |
|  |  | L-Leucine | 11.1 | g |
|  |  | L-Lysine-HCl | 18.0 | g |
|  |  | L-Phenylalanine | 7.5 | g |
|  |  | L-Proline | 3.5 | g |
|  |  | L-Serine | 3.5 | g |
|  |  | L-Threonine | 8.2 | g |
|  |  | L-Tryptophan | 1.8 | g |
|  |  | L-Tyrosine | 5.0 | g |
|  |  | L-Valine | 8.2 | g |
| **Carbohydrates** | | |  |  |
|  |  | Sucrose | 450.3 | g |
|  |  | Corn starch | 150 | g |
|  |  | Maltodextrin (Lodex 10) | 50 | g |
| **Fiber** | |  |  |  |
|  |  | Cellulose (Solka Floc, FCC200) | 30 | g |
| **Fat** |  |  |  |  |
|  |  | Corn oil | 100 | g |
| **Vitamins** | |  |  |  |
|  | Mix V10001: | |  |  |
|  |  | Sucrose | 14.6763 | g |
|  |  | Vit A Acetate, 500,000 IU/gm | 0.012 | g |
|  |  | Vit E Acetate, 500 IU/gm | 0.15 | g |
|  |  | Vit D3, 100,00 IU/gm | 15 | mg |
|  |  | Menadione Sodium Bisulfite | 1.2 | mg |
|  |  | Thiamin HCl (Vit B1) | 9 | mg |
|  |  | Riboflavin (Vit B2) | 9 | mg |
|  |  | Nicotinic Acid (Niacin B3) | 45 | mg |
|  |  | Calcium Pantothenate (B5) | 24 | mg |
|  |  | Pyridoxine-HCl (Vit B6) | 10.5 | mg |
|  |  | Biotin (B7), 1% | 30 | mg |
|  |  | Folic Acid (B9) | 3 | mg |
|  |  | Cyanocobalamin (Vit B12), 0.1% | 15 | mg |
| **Minerals (mix S10001)** | | |  |  |
|  |  | Sodium bicarbonate | 7.5 | g |
|  | Mix S10001: | |  |  |
|  |  | Sucrose, Fine Granulated | 4.13 | g |
|  |  | Calcium Phosphate, Dibasic | 17.5 | g |
|  |  | Potassium Citrate, Monohydrate | 7.7 | g |
|  |  | Sodium Chloride | 2.59 | g |
|  |  | Potassium Sulfate | 1.82 | g |
|  |  | Magnesium Oxide, Heavy, USP | 0.84 | g |
|  |  | Ferric Citrate | 0.21 | g |
|  |  | Manganese Carbonate Hydrate | 122.5 | mg |
|  |  | Zinc Carbonate | 56 | mg |
|  |  | Chromium Potassium Sulfate | 19.25 | mg |
|  |  | Copper Carbonate | 10.5 | mg |
|  |  | Potassium Iodate | 0.35 | mg |
|  |  | Sodium Selenite | 0.35 | mg |
| **Dye** |  |  |  |  |
|  |  | FD&C Blue Dye #1 | 0.05 | g |
| **Total** | |  | 1009.25 | g |

Table S3. **MetaCyc pathways and enzymes associated with liver fibrosis and advanced liver fibrosis in the human cohort.** A total of 8 pathways and 26 enzymes displayed a significant negative association (by both Mann-Whitney and logistic regression) with both liver fibrosis and advanced fibrosis. The four pathways and six enzymes in bold were found to be enriched in the stool of mice after bacterial inoculation. Median abundances are shown in copies per million (CPM). Adjusted odds ratios (AORs) were adjusted for age and gender. MW *p:* Mann-whitney *p*-value; FC: fold change between medians; AOR (low): risk with low abundance; AOR (high): risk with high abundance; *p*: logistic regression *p*-value.

|  |  | **Liver fibrosis** | | | | | | | | | **Advanced liver fibrosis** | | | | | | | |
| --- | --- | --- | --- | --- | --- | --- | --- | --- | --- | --- | --- | --- | --- | --- | --- | --- | --- | --- |
| **MetaCyc ID** | **Description** | **CPM**  **No fibrosis** | **CPM Fibrosis** | **MW *p*** | **FC** | **AOR (low)** | ***p*** | **AOR (high)** | ***p*** | **CPM**  **No advanced fibrosis** | | **CPM Advanced fibrosis** | **MW *p*** | **FC** | **AOR (low)** | ***p*** | **AOR (high)** | ***p*** |
| **Pathways** | | | | | | | | | | | | | | | | | | |
| CITRULBIO-PWY | L-citrulline biosynthesis | 403 | 173 | <0.001 | 0.43 | 4.33 (1.88-9.98) | 0.001 | 0.23 (0.10-0.53) | 0.001 | 375 | | 247 | 0.010 | 0.66 | 2.44 (1.11-5.37) | 0.027 | 0.36 (0.13-0.98) | 0.045 |
| **NONOXIPENT-PWY** | **pentose phosphate pathway (non-oxidative branch)** | **10178** | **9328** | **0.003** | **0.92** | **2.01 (1.06-3.81)** | **0.032** | **0.24 (0.08-0.70)** | **0.009** | **10115** | | **8952** | **0.007** | **0.89** | **3.13 (1.43-6.84)** | **0.004** | **0.30 (0.09-1.03)** | **0.056** |
| PWY-1042 | glycolysis IV (plant cytosol) | 13372 | 12591 | 0.021 | 0.94 | 2.44 (1.21-4.91) | 0.013 | 0.72 (0.33-1.59) | 0.418 | 13349 | | 11749 | 0.031 | 0.88 | 3.11 (1.39-6.96) | 0.006 | 0.76 (0.33-1.79) | 0.534 |
| PWY-4984 | urea cycle | 341 | 135 | <0.001 | 0.40 | 4.20 (1.82-9.66) | 0.001 | 0.24 (0.10-0.55) | 0.001 | 316 | | 207 | 0.010 | 0.66 | 2.84 (1.30-6.22) | 0.009 | 0.36 (0.13-0.98) | 0.045 |
| **PWY-5667** | **CDP-diacylglycerol biosynthesis I** | **12390** | **11698** | **0.006** | **0.94** | **1.85 (0.95-3.61)** | **0.073** | **0.30 (0.13-0.71)** | **0.006** | **12403** | | **11412** | **0.004** | **0.92** | **2.21 (1.00-4.89)** | **0.050** | **0.29 (0.10-0.86)** | **0.025** |
| PWY-5695 | urate biosynthesis/inosine 5'-phosphate degradation | 8411 | 7892 | 0.049 | 0.94 | 1.73 (0.90-3.31) | 0.100 | 0.39 (0.16-0.96) | 0.040 | 8404 | | 7476 | 0.028 | 0.89 | 1.99 (0.92-4.30) | 0.081 | 0.31 (0.09-1.04) | 0.058 |
| **PWY-6703** | **preQ0 biosynthesis** | **5374** | **4686** | **<0.001** | **0.87** | **3.25 (1.68-6.29)** | **<0.001** | **0.31 (0.13-0.72)** | **0.007** | **5221** | | **4764** | **0.019** | **0.91** | **2.74 (1.25-6.00)** | **0.012** | **0.45 (0.18-1.15)** | **0.095** |
| **PWY0-1319** | **CDP-diacylglycerol biosynthesis II** | **12390** | **11698** | **0.006** | **0.94** | **1.85 (0.95-3.61)** | **0.073** | **0.30 (0.13-0.71)** | **0.006** | **12403** | | **11412** | **0.004** | **0.92** | **2.21 (1.00-4.89)** | **0.050** | **0.29 (0.10-0.86)** | **0.025** |
| **Enzymes** | | | | | | | | | | | | | | | | | | |
| 1.1.1.18 | Inositol 2-dehydrogenase | 96 | 75 | 0.028 | 0.78 | 1.92 (0.99-3.73) | 0.055 | 0.59 (0.28-1.22) | 0.153 | 96 | | 72 | 0.044 | 0.75 | 2.34 (1.07-5.15) | 0.034 | 0.58 (0.24-1.40) | 0.225 |
| 1.1.1.38,4.1.1.3 | Malate dehydrogenase (oxaloacetate-decarboxylating)\| Oxaloacetate decarboxylase | 1081 | 865 | 0.005 | 0.80 | 2.11 (1.08-4.15) | 0.030 | 0.35 (0.16-0.79) | 0.011 | 1050 | | 833 | 0.018 | 0.79 | 2.21 (1.00-4.89) | 0.050 | 0.38 (0.14-1.03) | 0.057 |
| 1.1.3.15 | (S)-2-hydroxy-acid oxidase | 105 | 77 | 0.007 | 0.73 | 1.88 (1.00-3.55) | 0.052 | 0.39 (0.17-0.86) | 0.021 | 104 | | 77 | 0.045 | 0.74 | 2.00 (0.90-4.44) | 0.088 | 0.38 (0.14-1.03) | 0.057 |
| 1.14.13.39 | Nitric-oxide synthase (NADPH) | 122 | 102 | 0.011 | 0.84 | 1.90 (0.96-3.78) | 0.067 | 0.44 (0.20-0.95) | 0.036 | 123 | | 94 | 0.012 | 0.77 | 1.97 (0.90-4.34) | 0.090 | 0.40 (0.15-1.07) | 0.067 |
| 1.17.7.3 | (E)-4-hydroxy-3-methylbut-2-enyl-diphosphate synthase (flavodoxin) | 1341 | 1280 | 0.040 | 0.95 | 1.99 (1.05-3.78) | 0.036 | 0.71 (0.32-1.55) | 0.391 | 1337 | | 1194 | 0.001 | 0.89 | 3.06 (1.40-6.70) | 0.005 | 0.33 (0.10-1.13) | 0.078 |
| 1.2.7.1 | Pyruvate synthase | 94 | 75 | 0.047 | 0.80 | 1.52 (0.79-2.92) | 0.211 | 0.59 (0.26-1.32) | 0.199 | 94 | | 76 | 0.036 | 0.80 | 1.94 (0.89-4.22) | 0.097 | 0.59 (0.24-1.43) | 0.244 |
| **1.2.7.3** | **2-oxoglutarate synthase** | **98** | **69** | **0.008** | **0.71** | **2.37 (1.22-4.60)** | **0.011** | **0.30 (0.11-0.79)** | **0.015** | **94** | | **52** | **0.044** | **0.55** | **2.72 (1.24-5.94)** | **0.012** | **0.29 (0.09-1.01)** | **0.051** |
| 1.2.7.8 | Indolepyruvate ferredoxin oxidoreductase | 517 | 464 | 0.041 | 0.90 | 1.40 (0.70-2.80) | 0.337 | 0.51 (0.24-1.08) | 0.079 | 512 | | 405 | 0.015 | 0.79 | 1.66 (0.76-3.59) | 0.201 | 0.29 (0.10-0.86) | 0.025 |
| 1.2.99.2 | Carbon-monoxide dehydrogenase (acceptor) | 356 | 287 | 0.007 | 0.81 | 2.40 (1.26-4.56) | 0.007 | 0.59 (0.29-1.22) | 0.155 | 356 | | 294 | 0.032 | 0.83 | 2.34 (1.08-5.06) | 0.031 | 0.62 (0.23-1.70) | 0.352 |
| **2.5.1.47** | **Cysteine synthase** | **1294** | **1216** | **0.003** | **0.94** | **1.85 (0.94-3.64)** | **0.073** | **0.25 (0.09-0.73)** | **0.011** | **1287** | | **1152** | **0.004** | **0.90** | **2.70 (1.23-5.90)** | **0.013** | **0.29 (0.10-0.85)** | **0.024** |
| 2.7.1.11 | 6-phosphofructokinase | 2145 | 1955 | 0.002 | 0.91 | 2.41 (1.24-4.68) | 0.009 | 0.51 (0.24-1.08) | 0.079 | 2123 | | 1887 | 0.001 | 0.89 | 3.56 (1.61-7.88) | 0.002 | 0.50 (0.20-1.26) | 0.142 |
| 2.7.2.8 | Acetylglutamate kinase | 1330 | 1267 | 0.045 | 0.95 | 1.79 (0.94-3.40) | 0.077 | 0.64 (0.31-1.29) | 0.210 | 1324 | | 1239 | 0.002 | 0.94 | 3.07 (1.40-6.70) | 0.005 | 0.40 (0.15-1.08) | 0.069 |
| 2.7.7.41 | Phosphatidate cytidylyltransferase | 1064 | 902 | 0.012 | 0.85 | 2.57 (1.36-4.86) | 0.004 | 0.57 (0.28-1.18) | 0.128 | 1058 | | 866 | 0.049 | 0.82 | 2.77 (1.26-6.08) | 0.011 | 0.45 (0.15-1.33) | 0.149 |
| 3.2.1.21 | Beta-glucosidase | 1072 | 951 | 0.034 | 0.89 | 1.92 (1.01-3.63) | 0.046 | 0.49 (0.21-1.14) | 0.098 | 1079 | | 919 | 0.031 | 0.85 | 2.05 (0.95-4.43) | 0.069 | 0.46 (0.18-1.18) | 0.106 |
| 3.2.1.51 | Alpha-L-fucosidase | 124 | 85 | 0.011 | 0.69 | 2.20 (1.15-4.17) | 0.016 | 0.51 (0.24-1.08) | 0.080 | 122 | | 84 | 0.021 | 0.69 | 2.27 (1.05-4.93) | 0.038 | 0.38 (0.14-1.01) | 0.053 |
| 3.2.1.55 | Non-reducing end alpha-L-arabinofuranosidase | 213 | 163 | 0.014 | 0.77 | 1.87 (0.98-3.54) | 0.056 | 0.49 (0.23-1.04) | 0.063 | 212 | | 163 | 0.017 | 0.77 | 2.01 (0.93-4.33) | 0.076 | 0.39 (0.14-1.04) | 0.060 |
| 3.4.21.89 | Signal peptidase I | 2510 | 2313 | 0.005 | 0.92 | 2.26 (1.16-4.42) | 0.017 | 0.31 (0.13-0.71) | 0.006 | 2488 | | 2297 | 0.015 | 0.92 | 2.26 (1.03-4.99) | 0.043 | 0.20 (0.06-0.68) | 0.010 |
| **3.5.1.11** | **Penicillin amidase** | **169** | **55** | **<0.001** | **0.33** | **3.16 (1.65-6.02)** | **<0.001** | **0.26 (0.09-0.76)** | **0.013** | **162** | | **55** | **0.016** | **0.34** | **2.80 (1.27-6.16)** | **0.010** | **0.43 (0.15-1.29)** | **0.132** |
| 3.5.1.24 | Choloylglycine hydrolase | 283 | 224 | 0.002 | 0.79 | 2.45 (1.27-4.73) | 0.008 | 0.38 (0.17-0.86) | 0.019 | 284 | | 243 | 0.021 | 0.86 | 2.04 (0.92-4.53) | 0.081 | 0.37 (0.14-1.01) | 0.052 |
| 3.5.4.12 | dCMP deaminase | 309 | 255 | 0.020 | 0.83 | 2.29 (1.20-4.38) | 0.012 | 0.57 (0.25-1.28) | 0.175 | 307 | | 251 | 0.041 | 0.82 | 2.60 (1.19-5.66) | 0.016 | 0.46 (0.16-1.38) | 0.166 |
| **4.2.1.126** | **N-acetylmuramic acid 6-phosphate etherase** | **259** | **183** | **0.003** | **0.71** | **3.11 (1.61-6.00)** | **0.001** | **0.31 (0.12-0.83)** | **0.019** | **255** | | **199** | **0.041** | **0.78** | **3.03 (1.38-6.68)** | **0.006** | **0.46 (0.18-1.17)** | **0.103** |
| **4.6.1.12** | **2-C-methyl-D-erythritol 2,4-cyclodiphosphate synthase** | **1173** | **988** | **0.007** | **0.84** | **2.26 (1.19-4.29)** | **0.013** | **0.26 (0.10-0.63)** | **0.003** | **1165** | | **978** | **0.022** | **0.84** | **2.04 (0.94-4.41)** | **0.071** | **0.21 (0.06-0.71)** | **0.012** |
| 4.99.1.3 | Sirohydrochlorin cobaltochelatase | 92 | 74 | 0.021 | 0.80 | 1.81 (0.91-3.58) | 0.089 | 0.42 (0.19-0.91) | 0.029 | 91 | | 74 | 0.030 | 0.81 | 2.00 (0.88-4.50) | 0.096 | 0.39 (0.14-1.05) | 0.063 |
| **5.3.1.14** | **L-rhamnose isomerase** | **431** | **344** | **0.032** | **0.80** | **1.99 (1.05-3.79)** | **0.036** | **0.40 (0.19-0.88)** | **0.022** | **424** | | **267** | **0.009** | **0.63** | **2.80 (1.28-6.09)** | **0.010** | **0.38 (0.14-1.02)** | **0.054** |
| 6.3.5.1 | NAD(+) synthase (glutamine-hydrolyzing) | 195 | 169 | 0.019 | 0.87 | 1.91 (1.00-3.65) | 0.049 | 0.52 (0.25-1.11) | 0.091 | 198 | | 163 | 0.033 | 0.82 | 1.99 (0.92-4.30) | 0.082 | 0.58 (0.24-1.41) | 0.233 |
| 6.3.5.11 | Cobyrinate a,c-diamide synthase (glutamine-hydrolyzing) | 260 | 217 | 0.024 | 0.83 | 2.49 (1.31-4.73) | 0.005 | 0.61 (0.29-1.26) | 0.180 | 260 | | 189 | 0.021 | 0.73 | 3.61 (1.65-7.88) | 0.001 | 0.49 (0.19-1.25) | 0.134 |

Table S4. **MetaCyc pathways and enzymes associated with bacterial inoculation.** A total of 94 pathways and 328 enzymes absent in GF-MCD mice, detected in all three bacterial inocula, and detected in all GF-MCD-B mice at d14 were identified. Abundance is shown as copies per million (CPM). Pathways and enzymes with CPM <10 across all inocula and stool samples were excluded. The four pathways and six enzymes in bold were also found to be negatively associated with both liver fibrosis and advanced liver fibrosis in the human cohort.

|  |  | **Bacterial inocula** | | | **GF-MCD-B mice stool** | |
| --- | --- | --- | --- | --- | --- | --- |
| **Metacyc ID** | **Description** | **d0** | **d5** | **d10** | **d14** | **d28** |
| **Pathways** | | | | | | |
| ASPASN-PWY | superpathway of L-aspartate and L-asparagine biosynthesis | 5,530 | 7,304 | 6,572 | 9,621 | 9,250 |
| BRANCHED-CHAIN-AA-SYN-PWY | superpathway of branched amino acid biosynthesis | 2,268 | 6,405 | 1,684 | 2,517 | 86 |
| CALVIN-PWY | Calvin-Benson-Bassham cycle | 12,318 | 15,584 | 13,775 | 13,933 | 15,257 |
| COA-PWY-1 | coenzyme A biosynthesis II (mammalian) | 15,689 | 13,408 | 16,771 | 18,455 | 18,934 |
| DTDPRHAMSYN-PWY | dTDP-L-rhamnose biosynthesis I | 8,095 | 12,290 | 8,246 | 15,361 | 17,899 |
| GLYCOLYSIS | glycolysis I (from glucose 6-phosphate) | 9,851 | 10,632 | 6,787 | 15,022 | 10,388 |
| ILEUSYN-PWY | L-isoleucine biosynthesis I (from threonine) | 18,581 | 16,569 | 19,060 | 16,666 | 9,516 |
| NONMEVIPP-PWY | methylerythritol phosphate pathway I | 17,422 | 16,211 | 19,256 | 15,072 | 13,518 |
| **NONOXIPENT-PWY** | **pentose phosphate pathway (non-oxidative branch)** | **15,746** | **17,460** | **18,036** | **21,092** | **29,020** |
| PANTO-PWY | phosphopantothenate biosynthesis I | 15,990 | 13,820 | 16,957 | 16,928 | 17,029 |
| PEPTIDOGLYCANSYN-PWY | peptidoglycan biosynthesis I (meso-diaminopimelate containing) | 16,304 | 13,457 | 15,277 | 15,320 | 15,506 |
| PWY-1042 | glycolysis IV (plant cytosol) | 11,608 | 16,513 | 14,049 | 16,496 | 17,074 |
| PWY-1269 | CMP-3-deoxy-D-manno-octulosonate biosynthesis I | 9,763 | 10,567 | 10,253 | 13,180 | 13,687 |
| PWY-2942 | L-lysine biosynthesis III | 17,663 | 14,753 | 19,061 | 18,362 | 21,322 |
| PWY-3001 | superpathway of L-isoleucine biosynthesis I | 2,623 | 6,495 | 4,652 | 4,105 | 472 |
| PWY-5097 | L-lysine biosynthesis VI | 18,973 | 16,350 | 20,265 | 19,081 | 24,553 |
| PWY-5101 | L-isoleucine biosynthesis II | 914 | 1,039 | 1,071 | 1,559 | 63 |
| PWY-5103 | L-isoleucine biosynthesis III | 1,747 | 5,226 | 1,288 | 1,948 | 65 |
| PWY-5484 | glycolysis II (from fructose 6-phosphate) | 9,647 | 10,622 | 6,814 | 14,879 | 10,111 |
| PWY-5659 | GDP-mannose biosynthesis | 5,277 | 7,643 | 6,282 | 9,424 | 4,787 |
| **PWY-5667** | **CDP-diacylglycerol biosynthesis I** | **13,853** | **14,082** | **17,768** | **19,219** | **22,433** |
| PWY-5686 | UMP biosynthesis | 21,047 | 20,123 | 22,828 | 22,517 | 25,741 |
| PWY-5695 | urate biosynthesis/inosine 5'-phosphate degradation | 13,785 | 11,574 | 13,192 | 14,494 | 16,959 |
| PWY-5973 | cis-vaccenate biosynthesis | 11,243 | 8,425 | 11,241 | 5,885 | 4,064 |
| PWY-6121 | 5-aminoimidazole ribonucleotide biosynthesis I | 12,737 | 11,074 | 12,490 | 12,756 | 12,837 |
| PWY-6122 | 5-aminoimidazole ribonucleotide biosynthesis II | 12,077 | 10,442 | 11,289 | 12,058 | 11,338 |
| PWY-6125 | superpathway of guanosine nucleotides de novo biosynthesis II | 9,624 | 8,246 | 7,395 | 3,237 | 4,182 |
| PWY-6126 | superpathway of adenosine nucleotides de novo biosynthesis II | 10,413 | 14,018 | 9,083 | 8,029 | 8,132 |
| PWY-6147 | 6-hydroxymethyl-dihydropterin diphosphate biosynthesis I | 4,238 | 5,300 | 1,523 | 9,252 | 4,011 |
| PWY-6151 | S-adenosyl-L-methionine cycle I | 3,453 | 4,009 | 6,628 | 9,637 | 3,153 |
| PWY-6277 | superpathway of 5-aminoimidazole ribonucleotide biosynthesis | 12,077 | 10,442 | 11,289 | 12,058 | 11,338 |
| PWY-6386 | UDP-N-acetylmuramoyl-pentapeptide biosynthesis II (lysine-containing) | 18,186 | 16,129 | 19,820 | 18,528 | 20,253 |
| PWY-6387 | UDP-N-acetylmuramoyl-pentapeptide biosynthesis I (meso-diaminopimelate containing) | 17,234 | 14,908 | 18,241 | 16,630 | 17,698 |
| PWY-6545 | pyrimidine deoxyribonucleotides de novo biosynthesis III | 5,939 | 5,158 | 4,698 | 1,794 | 218 |
| PWY-6609 | adenine and adenosine salvage III | 10,682 | 10,002 | 9,078 | 12,343 | 13,610 |
| PWY-6700 | queuosine biosynthesis | 16,026 | 13,451 | 14,940 | 13,819 | 17,232 |
| **PWY-6703** | **preQ0 biosynthesis** | **19,520** | **12,502** | **20,281** | **18,983** | **26,619** |
| PWY-6737 | starch degradation V | 6,042 | 7,248 | 5,098 | 2,048 | 271 |
| PWY-7111 | pyruvate fermentation to isobutanol (engineered) | 18,581 | 16,569 | 19,060 | 16,666 | 21,987 |
| PWY-7184 | pyrimidine deoxyribonucleotides de novo biosynthesis I | 7,251 | 6,224 | 5,401 | 2,426 | 3,001 |
| PWY-7187 | pyrimidine deoxyribonucleotides de novo biosynthesis II | 7,197 | 5,941 | 6,137 | 2,604 | 256 |
| PWY-7208 | superpathway of pyrimidine nucleobases salvage | 3,658 | 3,465 | 3,734 | 1,631 | 812 |
| PWY-7219 | adenosine ribonucleotides de novo biosynthesis | 24,886 | 22,318 | 23,897 | 26,994 | 33,512 |
| PWY-7220 | adenosine deoxyribonucleotides de novo biosynthesis II | 6,270 | 10,434 | 5,292 | 4,826 | 4,726 |
| PWY-7222 | guanosine deoxyribonucleotides de novo biosynthesis II | 6,270 | 10,434 | 5,292 | 4,826 | 4,726 |
| PWY-7228 | superpathway of guanosine nucleotides de novo biosynthesis I | 12,521 | 8,163 | 7,878 | 2,967 | 3,956 |
| PWY-7229 | superpathway of adenosine nucleotides de novo biosynthesis I | 12,230 | 15,564 | 10,350 | 9,570 | 9,696 |
| PWY-724 | superpathway of L-lysine, L-threonine and L-methionine biosynthesis II | 6,024 | 9,390 | 9,188 | 6,997 | 2,982 |
| PWY-7663 | gondoate biosynthesis (anaerobic) | 15,524 | 13,921 | 16,429 | 17,876 | 22,742 |
| **PWY0-1319** | **CDP-diacylglycerol biosynthesis II** | **13,853** | **14,082** | **17,768** | **19,219** | **22,433** |
| PWY0-166 | superpathway of pyrimidine deoxyribonucleotides de novo biosynthesis (E. coli) | 7,575 | 6,139 | 6,115 | 2,477 | 254 |
| PWY66-400 | glycolysis VI (metazoan) | 8,152 | 11,786 | 5,726 | 13,041 | 8,734 |
| PYRIDNUCSYN-PWY | NAD biosynthesis I (from aspartate) | 1,007 | 1,715 | 2,023 | 1,035 | 514 |
| SER-GLYSYN-PWY | superpathway of L-serine and glycine biosynthesis I | 132 | 373 | 638 | 1,193 | 102 |
| THISYNARA-PWY | superpathway of thiamin diphosphate biosynthesis III (eukaryotes) | 2,955 | 3,988 | 1,786 | 6,884 | 3,526 |
| THRESYN-PWY | superpathway of L-threonine biosynthesis | 3,964 | 7,059 | 6,423 | 4,880 | 1,879 |
| TRNA-CHARGING-PWY | tRNA charging | 14,624 | 13,378 | 14,011 | 12,779 | 14,955 |
| VALSYN-PWY | L-valine biosynthesis | 18,581 | 16,569 | 19,060 | 16,666 | 21,987 |
| 1CMET2-PWY | N10-formyl-tetrahydrofolate biosynthesis | 15,346 | 10,391 | 13,379 | 12,073 | 14,182 |
| ANAEROFRUCAT-PWY | homolactic fermentation | 8,343 | 9,944 | 6,848 | 4,675 | 6,755 |
| ANAGLYCOLYSIS-PWY | glycolysis III (from glucose) | 10,679 | 13,596 | 9,608 | 13,990 | 11,454 |
| ARGININE-SYN4-PWY | L-ornithine de novo biosynthesis | 7,396 | 7,586 | 8,732 | 11,647 | 13,378 |
| DENOVOPURINE2-PWY | superpathway of purine nucleotides de novo biosynthesis II | 8,573 | 8,436 | 7,456 | 4,095 | 4,216 |
| GLCMANNANAUT-PWY | superpathway of N-acetylglucosamine, N-acetylmannosamine and N-acetylneuraminate degradation | 6,458 | 6,141 | 3,989 | 4,282 | 173 |
| HISDEG-PWY | L-histidine degradation I | 9,553 | 9,493 | 9,787 | 15,331 | 3,663 |
| HISTSYN-PWY | L-histidine biosynthesis | 6,747 | 6,841 | 7,668 | 7,169 | 9,478 |
| HOMOSER-METSYN-PWY | L-methionine biosynthesis I | 3,744 | 4,375 | 5,077 | 6,568 | 7,743 |
| HSERMETANA-PWY | L-methionine biosynthesis III | 2,851 | 4,513 | 4,374 | 2,899 | 1,070 |
| MET-SAM-PWY | superpathway of S-adenosyl-L-methionine biosynthesis | 6,188 | 7,176 | 7,975 | 8,385 | 8,279 |
| METSYN-PWY | L-homoserine and L-methionine biosynthesis | 5,628 | 6,751 | 7,443 | 8,638 | 8,217 |
| PANTOSYN-PWY | pantothenate and coenzyme A biosynthesis I | 4,830 | 6,576 | 6,066 | 9,115 | 7,991 |
| PWY-3841 | folate transformations II | 16,063 | 10,814 | 14,095 | 12,773 | 14,878 |
| PWY-4242 | pantothenate and coenzyme A biosynthesis III | 2,492 | 3,897 | 3,126 | 5,733 | 4,643 |
| PWY-5030 | L-histidine degradation III | 875 | 1,165 | 1,541 | 1,747 | 210 |
| PWY-5347 | superpathway of L-methionine biosynthesis (transsulfuration) | 5,896 | 6,989 | 7,720 | 9,080 | 8,859 |
| PWY-6123 | inosine-5'-phosphate biosynthesis I | 7,891 | 8,300 | 8,484 | 7,986 | 8,625 |
| PWY-6124 | inosine-5'-phosphate biosynthesis II | 6,981 | 7,397 | 7,557 | 7,044 | 7,523 |
| PWY-6168 | flavin biosynthesis III (fungi) | 6,784 | 5,885 | 7,862 | 6,193 | 8,277 |
| PWY-6385 | peptidoglycan biosynthesis III (mycobacteria) | 15,647 | 13,375 | 14,970 | 15,633 | 16,388 |
| PWY-7198 | pyrimidine deoxyribonucleotides de novo biosynthesis IV | 5,829 | 4,921 | 4,759 | 1,737 | 181 |
| PWY-7199 | pyrimidine deoxyribonucleosides salvage | 373 | 586 | 453 | 1,953 | 121 |
| PWY-7210 | pyrimidine deoxyribonucleotides biosynthesis from CTP | 5,691 | 5,108 | 4,512 | 2,038 | 232 |
| PWY-7211 | superpathway of pyrimidine deoxyribonucleotides de novo biosynthesis | 5,177 | 6,415 | 5,772 | 2,467 | 606 |
| PWY-7282 | 4-amino-2-methyl-5-phosphomethylpyrimidine biosynthesis (yeast) | 4,650 | 5,561 | 3,409 | 10,012 | 6,007 |
| PWY-7323 | superpathway of GDP-mannose-derived O-antigen building blocks biosynthesis | 2,475 | 2,624 | 1,820 | 4,558 | 3,801 |
| PWY-7539 | 6-hydroxymethyl-dihydropterin diphosphate biosynthesis III (Chlamydia) | 4,090 | 4,930 | 1,499 | 8,477 | 3,939 |
| PWY-841 | superpathway of purine nucleotides de novo biosynthesis I | 9,231 | 8,549 | 7,943 | 4,086 | 4,239 |
| PWY0-1586 | peptidoglycan maturation (meso-diaminopimelate containing) | 10,497 | 6,280 | 11,719 | 13,734 | 27,590 |
| PWY0-162 | superpathway of pyrimidine ribonucleotides de novo biosynthesis | 4,146 | 6,895 | 6,326 | 2,587 | 1,460 |
| PWY0-845 | superpathway of pyridoxal 5'-phosphate biosynthesis and salvage | 18,788 | 14,146 | 20,422 | 20,480 | 26,676 |
| PYRIDOXSYN-PWY | pyridoxal 5'-phosphate biosynthesis I | 19,009 | 15,724 | 21,858 | 21,512 | 25,239 |
| RHAMCAT-PWY | L-rhamnose degradation I | 9,148 | 11,437 | 11,045 | 13,174 | 17,381 |
| RIBOSYN2-PWY | flavin biosynthesis I (bacteria and plants) | 5,657 | 5,382 | 6,493 | 5,802 | 7,531 |
| TRPSYN-PWY | L-tryptophan biosynthesis | 9,948 | 8,201 | 10,497 | 10,315 | 16,271 |
| **Enzymes** | | | | | | |
| 1.1.1.133 | dTDP-4-dehydrorhamnose reductase | 401 | 567 | 379 | 709 | 760 |
| 1.1.1.169 | 2-dehydropantoate 2-reductase | 1,070 | 820 | 1,133 | 956 | 944 |
| 1.1.1.17 | Mannitol-1-phosphate 5-dehydrogenase | 91 | 233 | 202 | 72 | 29 |
| 1.1.1.22 | UDP-glucose 6-dehydrogenase | 355 | 451 | 396 | 551 | 568 |
| 1.1.1.23 | Histidinol dehydrogenase | 1,007 | 941 | 986 | 769 | 628 |
| 1.1.1.262 | 4-hydroxythreonine-4-phosphate dehydrogenase | 1,519 | 1,570 | 1,514 | 1,505 | 1,528 |
| 1.1.1.267 | 1-deoxy-D-xylulose-5-phosphate reductoisomerase | 1,067 | 1,349 | 995 | 796 | 788 |
| 1.1.1.271 | GDP-L-fucose synthase | 1,704 | 1,163 | 1,576 | 1,085 | 1,737 |
| 1.1.1.29 | Glycerate dehydrogenase | 401 | 488 | 442 | 634 | 693 |
| 1.1.1.290 | 4-phosphoerythronate dehydrogenase | 1,415 | 1,304 | 1,412 | 1,151 | 1,330 |
| 1.1.1.3 | Homoserine dehydrogenase | 456 | 798 | 537 | 402 | 211 |
| 1.1.1.58 | Tagaturonate reductase | 490 | 556 | 673 | 670 | 948 |
| 1.1.1.69 | Gluconate 5-dehydrogenase | 702 | 814 | 773 | 904 | 1,165 |
| 1.1.1.77 | Lactaldehyde reductase | 604 | 871 | 677 | 749 | 751 |
| 1.1.1.85 | 3-isopropylmalate dehydrogenase | 1,093 | 1,056 | 1,146 | 1,009 | 1,086 |
| 1.1.1.86 | Ketol-acid reductoisomerase (NADP(+)) | 1,380 | 1,617 | 1,524 | 1,627 | 1,655 |
| 1.1.1.94 | Glycerol-3-phosphate dehydrogenase (NAD(P)(+)) | 1,785 | 1,555 | 1,471 | 1,200 | 1,401 |
| 1.10.3.10 | Ubiquinol oxidase (H(+)-transporting) | 29 | 77 | 75 | 24 | 9 |
| 1.11.1.1 | NADH peroxidase | 48 | 50 | 55 | 49 | 73 |
| 1.14.13.81 | Magnesium-protoporphyrin IX monomethyl ester (oxidative) cyclase | 56 | 148 | 74 | 70 | 52 |
| 1.17.1.2 | 4-hydroxy-3-methylbut-2-enyl diphosphate reductase | 850 | 858 | 1,041 | 641 | 304 |
| 1.17.1.8 | 4-hydroxy-tetrahydrodipicolinate reductase | 1,131 | 1,165 | 1,223 | 1,153 | 837 |
| 1.17.4.2 | Ribonucleoside-triphosphate reductase | 397 | 648 | 377 | 437 | 378 |
| 1.17.7.3 | (E)-4-hydroxy-3-methylbut-2-enyl-diphosphate synthase (flavodoxin) | 1,164 | 963 | 1,150 | 1,033 | 890 |
| 1.2.1.11 | Aspartate-semialdehyde dehydrogenase | 1,194 | 1,140 | 1,267 | 1,149 | 1,454 |
| 1.2.1.38 | N-acetyl-gamma-glutamyl-phosphate reductase | 1,135 | 920 | 993 | 692 | 761 |
| 1.2.1.41 | Glutamate-5-semialdehyde dehydrogenase | 1,667 | 1,393 | 1,707 | 1,589 | 2,002 |
| **1.2.7.3** | **2-oxoglutarate synthase** | **569** | **698** | **670** | **689** | **798** |
| 1.2.7.7 | 3-methyl-2-oxobutanoate dehydrogenase (ferredoxin) | 21 | 18 | 26 | 22 | 21 |
| 1.2.7.8 | Indolepyruvate ferredoxin oxidoreductase | 811 | 576 | 593 | 239 | 150 |
| 1.3.1.12 | Prephenate dehydrogenase | 18 | 88 | 19 | 29 | 2 |
| 1.3.1.14 | Dihydroorotate dehydrogenase (NAD(+)) | 930 | 690 | 651 | 632 | 880 |
| 1.3.1.9 | Enoyl-[acyl-carrier-protein] reductase (NADH) | 1,119 | 984 | 964 | 1,048 | 1,164 |
| 1.3.1.98 | UDP-N-acetylmuramate dehydrogenase | 1,147 | 1,242 | 1,313 | 1,142 | 739 |
| 1.3.5.1 | Succinate dehydrogenase (quinone) | 187 | 232 | 235 | 187 | 151 |
| 1.3.99.22 | Coproporphyrinogen dehydrogenase | 341 | 586 | 390 | 439 | 189 |
| 1.3.99.5 | 3-oxo-5-alpha-steroid 4-dehydrogenase (acceptor) | 271 | 463 | 264 | 386 | 210 |
| 1.4.1.1 | Alanine dehydrogenase | 668 | 574 | 392 | 608 | 262 |
| 1.4.1.13 | Glutamate synthase (NADPH) | 453 | 697 | 577 | 395 | 203 |
| 1.4.1.14 | Glutamate synthase (NADH) | 26 | 26 | 42 | 37 | 34 |
| 1.4.1.16 | Diaminopimelate dehydrogenase | 998 | 725 | 973 | 776 | 922 |
| 1.4.1.2 | Glutamate dehydrogenase | 206 | 240 | 284 | 316 | 330 |
| 1.4.1.3 | Glutamate dehydrogenase (NAD(P)(+)) | 307 | 389 | 352 | 522 | 458 |
| 1.4.3.16 | L-aspartate oxidase | 740 | 682 | 868 | 791 | 742 |
| 1.4.3.5 | Pyridoxal 5'-phosphate synthase | 1,225 | 955 | 1,231 | 1,005 | 1,180 |
| 1.4.4.2 | Glycine dehydrogenase (aminomethyl-transferring) | 1,378 | 1,542 | 1,762 | 1,435 | 1,401 |
| 1.5.1.5 | Methylenetetrahydrofolate dehydrogenase (NADP(+)) | 779 | 471 | 598 | 462 | 472 |
| 1.5.1.7 | Saccharopine dehydrogenase (NAD(+), L-lysine-forming) | 49 | 45 | 35 | 32 | 18 |
| 1.6.5.11 | NADH dehydrogenase (quinone) | 4,719 | 5,782 | 6,564 | 6,916 | 7,391 |
| 1.6.5.8 | NADH:ubiquinone reductase (Na(+)-transporting) | 6,283 | 6,364 | 7,210 | 6,487 | 7,124 |
| 1.7.1.13 | PreQ(1) synthase | 1,016 | 809 | 1,000 | 745 | 1,008 |
| 1.7.2.2 | Nitrite reductase (cytochrome; ammonia-forming) | 1,344 | 912 | 848 | 1,023 | 918 |
| 1.7.99.1 | Hydroxylamine reductase | 1,180 | 926 | 1,051 | 1,147 | 1,160 |
| 1.8.1.8 | Protein-disulfide reductase | 18 | 20 | 39 | 46 | 27 |
| 1.8.4.2 | Protein-disulfide reductase (glutathione) | 444 | 228 | 172 | 75 | 46 |
| 1.97.1.4 | [Formate-C-acetyltransferase]-activating enzyme | 1,075 | 979 | 925 | 936 | 604 |
| 2.1.1.13 | Methionine synthase | 180 | 208 | 201 | 239 | 273 |
| 2.1.1.131 | Precorrin-3B C(17)-methyltransferase | 2 | 4 | 16 | 18 | 1 |
| 2.1.1.163 | Demethylmenaquinone methyltransferase | 873 | 789 | 778 | 662 | 642 |
| 2.1.1.171 | 16S rRNA (guanine(966)-N(2))-methyltransferase | 6 | 9 | 18 | 29 | 8 |
| 2.1.1.181 | 23S rRNA (adenine(1618)-N(6))-methyltransferase | 593 | 514 | 572 | 770 | 1,192 |
| 2.1.1.182 | 16S rRNA (adenine(1518)-N(6)/adenine(1519)-N(6))-dimethyltransferase | 1,202 | 982 | 1,306 | 1,018 | 978 |
| 2.1.1.193 | 16S rRNA (uracil(1498)-N(3))-methyltransferase | 997 | 790 | 945 | 614 | 932 |
| 2.1.1.197 | Malonyl-[acyl-carrier protein] O-methyltransferase | 805 | 540 | 578 | 698 | 392 |
| 2.1.1.198 | 16S rRNA (cytidine(1402)-2'-O)-methyltransferase | 542 | 491 | 539 | 432 | 395 |
| 2.1.1.199 | 16S rRNA (cytosine(1402)-N(4))-methyltransferase | 1,228 | 1,139 | 1,073 | 776 | 821 |
| 2.1.1.223 | tRNA(1)(Val) (adenine(37)-N(6))-methyltransferase | 1,143 | 891 | 1,117 | 907 | 1,229 |
| 2.1.1.297 | Peptide chain release factor N(5)-glutamine methyltransferase | 1,184 | 831 | 1,092 | 694 | 1,301 |
| 2.1.1.37 | DNA (cytosine-5-)-methyltransferase | 1,571 | 1,596 | 1,425 | 856 | 250 |
| 2.1.1.72 | Site-specific DNA-methyltransferase (adenine-specific) | 798 | 763 | 925 | 989 | 1,901 |
| 2.1.2.11 | 3-methyl-2-oxobutanoate hydroxymethyltransferase | 1,139 | 996 | 1,082 | 899 | 990 |
| 2.1.2.3 | Phosphoribosylaminoimidazolecarboxamide formyltransferase | 417 | 459 | 522 | 564 | 685 |
| 2.1.2.5 | Glutamate formimidoyltransferase | 5 | 12 | 25 | 46 | 5 |
| 2.1.3.11 | N-succinylornithine carbamoyltransferase | 383 | 335 | 477 | 457 | 768 |
| 2.1.3.2 | Aspartate carbamoyltransferase | 1,297 | 1,223 | 1,317 | 1,139 | 1,323 |
| 2.1.3.3 | Ornithine carbamoyltransferase | 189 | 231 | 175 | 57 | 6 |
| 2.1.3.9 | N-acetylornithine carbamoyltransferase | 427 | 434 | 567 | 505 | 787 |
| 2.2.1.7 | 1-deoxy-D-xylulose-5-phosphate synthase | 1,796 | 1,806 | 2,083 | 2,165 | 1,718 |
| 2.2.1.9 | synthase | 684 | 511 | 609 | 264 | 291 |
| 2.3.1.117 | 2,3,4,5-tetrahydropyridine-2,6-dicarboxylate N-succinyltransferase | 47 | 79 | 76 | 44 | 9 |
| 2.3.1.128 | Ribosomal-protein-alanine N-acetyltransferase | 23 | 24 | 15 | 90 | 20 |
| 2.3.1.129 | Acyl-[acyl-carrier-protein]--UDP-N-acetylglucosamine O-acyltransferase | 2,702 | 2,591 | 3,177 | 2,986 | 3,007 |
| 2.3.1.180 | Beta-ketoacyl-[acyl-carrier-protein] synthase III | 2,095 | 2,086 | 2,290 | 2,241 | 2,231 |
| 2.3.1.182 | (R)-citramalate synthase | 37 | 36 | 79 | 103 | 62 |
| 2.3.1.30 | Serine O-acetyltransferase | 560 | 647 | 574 | 640 | 758 |
| 2.3.1.31 | Homoserine O-acetyltransferase | 127 | 263 | 224 | 86 | 20 |
| 2.3.1.46 | Homoserine O-succinyltransferase | 1,404 | 1,294 | 1,383 | 1,177 | 1,385 |
| 2.3.1.47 | 8-amino-7-oxononanoate synthase | 121 | 238 | 79 | 316 | 118 |
| 2.3.1.50 | Serine C-palmitoyltransferase | 32 | 40 | 175 | 182 | 42 |
| 2.3.1.54 | Formate C-acetyltransferase | 86 | 192 | 76 | 66 | 66 |
| 2.3.1.79 | Maltose O-acetyltransferase | 540 | 799 | 410 | 1,062 | 465 |
| 2.3.2.6 | Leucyltransferase | 52 | 98 | 383 | 447 | 57 |
| 2.4.1.1 | Glycogen phosphorylase | 792 | 973 | 642 | 588 | 675 |
| 2.4.1.11 | Glycogen(starch) synthase | 23 | 44 | 36 | 22 | 16 |
| 2.4.1.129 | Peptidoglycan glycosyltransferase | 860 | 938 | 859 | 1,211 | 1,393 |
| 2.4.1.18 | 1,4-alpha-glucan branching enzyme | 1,352 | 1,151 | 1,204 | 866 | 985 |
| 2.4.1.182 | Lipid-A-disaccharide synthase | 84 | 121 | 67 | 150 | 91 |
| 2.4.1.21 | Starch synthase | 539 | 497 | 332 | 47 | 34 |
| 2.4.1.281 | 4-O-beta-D-mannosyl-D-glucose phosphorylase | 1,688 | 1,808 | 1,400 | 2,243 | 2,152 |
| 2.4.1.57 | Phosphatidylinositol alpha-mannosyltransferase | 173 | 92 | 231 | 138 | 608 |
| 2.4.2.18 | Anthranilate phosphoribosyltransferase | 1,003 | 682 | 918 | 677 | 846 |
| 2.4.2.21 | Nicotinate-nucleotide--dimethylbenzimidazole phosphoribosyltransferase | 788 | 557 | 418 | 455 | 163 |
| 2.4.2.22 | Xanthine phosphoribosyltransferase | 976 | 714 | 672 | 600 | 759 |
| 2.4.2.9 | Uracil phosphoribosyltransferase | 148 | 176 | 183 | 106 | 23 |
| 2.4.99.17 | S-adenosylmethionine:tRNA ribosyltransferase-isomerase | 1,805 | 1,525 | 1,567 | 1,249 | 1,148 |
| 2.5.1.15 | Dihydropteroate synthase | 418 | 585 | 380 | 642 | 468 |
| 2.5.1.17 | Cob(I)yrinic acid a,c-diamide adenosyltransferase | 5 | 9 | 27 | 59 | 3 |
| 2.5.1.19 | 3-phosphoshikimate 1-carboxyvinyltransferase | 1,527 | 1,165 | 1,525 | 1,257 | 1,688 |
| 2.5.1.3 | Thiamine-phosphate diphosphorylase | 1,467 | 1,117 | 1,326 | 1,489 | 1,652 |
| **2.5.1.47** | **Cysteine synthase** | **1,666** | **1,753** | **1,645** | **1,643** | **1,647** |
| 2.5.1.48 | Cystathionine gamma-synthase | 211 | 311 | 301 | 363 | 421 |
| 2.5.1.49 | O-acetylhomoserine aminocarboxypropyltransferase | 744 | 991 | 971 | 1,107 | 1,179 |
| 2.5.1.54 | 3-deoxy-7-phosphoheptulonate synthase | 262 | 534 | 209 | 69 | 24 |
| 2.5.1.72 | Quinolinate synthase | 1,199 | 1,014 | 1,153 | 1,001 | 1,092 |
| 2.5.1.74 | 1,4-dihydroxy-2-naphthoate polyprenyltransferase | 1,566 | 1,469 | 1,810 | 1,579 | 1,727 |
| 2.5.1.75 | tRNA dimethylallyltransferase | 2,436 | 1,760 | 2,236 | 2,007 | 2,129 |
| 2.5.1.78 | 6,7-dimethyl-8-ribityllumazine synthase | 1,049 | 643 | 843 | 561 | 358 |
| 2.5.1.9 | Riboflavin synthase | 157 | 207 | 218 | 155 | 204 |
| 2.6.1.11 | Acetylornithine transaminase | 299 | 214 | 222 | 77 | 104 |
| 2.6.1.37 | 2-aminoethylphosphonate--pyruvate transaminase | 1,410 | 1,349 | 1,364 | 1,168 | 1,437 |
| 2.6.1.62 | Adenosylmethionine--8-amino-7-oxononanoate transaminase | 612 | 306 | 348 | 227 | 187 |
| 2.6.1.83 | LL-diaminopimelate aminotransferase | 1,631 | 1,355 | 1,462 | 1,186 | 1,449 |
| 2.6.1.85 | Aminodeoxychorismate synthase | 9 | 25 | 5 | 52 | 11 |
| 2.6.1.9 | Histidinol-phosphate transaminase | 951 | 629 | 903 | 631 | 530 |
| 2.6.99.2 | Pyridoxine 5'-phosphate synthase | 1,362 | 1,225 | 1,492 | 1,467 | 1,588 |
| 2.7.1.130 | Tetraacyldisaccharide 4'-kinase | 1,263 | 977 | 1,253 | 765 | 1,226 |
| 2.7.1.148 | 4-(cytidine 5'-diphospho)-2-C-methyl-D-erythritol kinase | 1,167 | 1,100 | 1,227 | 875 | 923 |
| 2.7.1.16 | Ribulokinase | 835 | 940 | 932 | 970 | 754 |
| 2.7.1.17 | Xylulokinase | 410 | 576 | 477 | 625 | 585 |
| 2.7.1.26 | Riboflavin kinase | 1,344 | 1,150 | 1,413 | 1,203 | 1,451 |
| 2.7.1.35 | Pyridoxal kinase | 1,447 | 983 | 1,240 | 1,181 | 1,644 |
| 2.7.1.39 | Homoserine kinase | 98 | 242 | 171 | 83 | 13 |
| 2.7.1.5 | Rhamnulokinase | 466 | 638 | 571 | 639 | 728 |
| 2.7.1.52 | Fucokinase | 3 | 2 | 7 | 11 | 3 |
| 2.7.1.71 | Shikimate kinase | 1,162 | 1,171 | 1,225 | 1,139 | 573 |
| 2.7.1.90 | Diphosphate--fructose-6-phosphate 1-phosphotransferase | 264 | 362 | 178 | 16 | 6 |
| 2.7.2.1 | Acetate kinase | 1,703 | 1,513 | 1,593 | 1,486 | 1,765 |
| 2.7.2.11 | Glutamate 5-kinase | 1,395 | 1,129 | 1,351 | 1,058 | 1,532 |
| 2.7.2.7 | Butyrate kinase | 586 | 949 | 995 | 892 | 502 |
| 2.7.2.8 | Acetylglutamate kinase | 1,337 | 1,154 | 1,308 | 1,225 | 1,293 |
| 2.7.3.9 | Phosphoenolpyruvate--protein phosphotransferase | 173 | 302 | 226 | 75 | 17 |
| 2.7.4.1 | Polyphosphate kinase | 2,266 | 2,099 | 2,323 | 2,282 | 3,451 |
| 2.7.4.16 | Thiamine-phosphate kinase | 1,184 | 1,333 | 1,215 | 1,123 | 1,050 |
| 2.7.4.7 | Phosphomethylpyrimidine kinase | 96 | 191 | 56 | 204 | 88 |
| 2.7.6.2 | Thiamine diphosphokinase | 263 | 476 | 103 | 508 | 227 |
| 2.7.6.3 | 2-amino-4-hydroxy-6-hydroxymethyldihydropteridine diphosphokinase | 174 | 262 | 57 | 320 | 117 |
| 2.7.6.5 | GTP diphosphokinase | 22 | 41 | 47 | 31 | 21 |
| 2.7.7.13 | Mannose-1-phosphate guanylyltransferase | 69 | 94 | 48 | 133 | 96 |
| 2.7.7.22 | Mannose-1-phosphate guanylyltransferase (GDP) | 577 | 700 | 622 | 925 | 931 |
| 2.7.7.38 | 3-deoxy-manno-octulosonate cytidylyltransferase | 1,371 | 1,169 | 1,567 | 1,123 | 1,241 |
| 2.7.7.60 | 2-C-methyl-D-erythritol 4-phosphate cytidylyltransferase | 2,025 | 1,408 | 1,799 | 926 | 1,223 |
| 2.7.7.63 | Lipoate--protein ligase | 39 | 71 | 17 | 93 | 25 |
| 2.7.7.72 | CCA tRNA nucleotidyltransferase | 7 | 9 | 5 | 11 | 11 |
| 2.7.7.8 | Polyribonucleotide nucleotidyltransferase | 1,243 | 1,227 | 1,116 | 1,069 | 1,049 |
| 2.7.8.13 | Phospho-N-acetylmuramoyl-pentapeptide-transferase | 1,203 | 959 | 1,057 | 897 | 760 |
| 2.7.8.26 | Adenosylcobinamide-GDP ribazoletransferase | 977 | 815 | 692 | 877 | 313 |
| 2.7.8.6 | Undecaprenyl-phosphate galactose phosphotransferase | 124 | 275 | 177 | 426 | 217 |
| 2.7.8.8 | CDP-diacylglycerol--serine O-phosphatidyltransferase | 238 | 259 | 133 | 370 | 357 |
| 2.7.9.1 | Pyruvate, phosphate dikinase | 279 | 415 | 281 | 138 | 115 |
| 2.7.9.2 | Pyruvate, water dikinase | 168 | 276 | 104 | 433 | 173 |
| 2.8.1.10 | Thiazole synthase | 510 | 559 | 715 | 822 | 866 |
| 2.8.1.6 | Biotin synthase | 749 | 391 | 330 | 192 | 144 |
| 2.8.3.1, 2.8.3.8 | Propionate CoA-transferase\|Acetate CoA-transferase | 47 | 113 | 85 | 29 | 7 |
| 2.8.3.8 | Acetate CoA-transferase | 47 | 113 | 85 | 29 | 7 |
| 2.8.4.3 | tRNA-2-methylthio-N(6)-dimethylallyladenosine synthase | 803 | 556 | 594 | 437 | 359 |
| 2.8.4.4 | [Ribosomal protein S12] (aspartate(89)-C(3))-methylthiotransferase | 1,055 | 947 | 1,060 | 830 | 688 |
| 3.1.1.11 | Pectinesterase | 3,339 | 4,903 | 3,831 | 4,194 | 3,837 |
| 3.1.1.96 | D-aminoacyl-tRNA deacylase | 1,173 | 1,029 | 1,195 | 927 | 963 |
| 3.1.11.5 | Exodeoxyribonuclease V | 50 | 115 | 104 | 42 | 10 |
| 3.1.2.23 | 4-hydroxybenzoyl-CoA thioesterase | 4 | 8 | 19 | 12 | 7 |
| 3.1.21.3 | Type I site-specific deoxyribonuclease | 776 | 1,028 | 1,010 | 615 | 770 |
| 3.1.22.4 | Crossover junction endodeoxyribonuclease | 1,169 | 1,219 | 1,172 | 717 | 573 |
| 3.1.26.11 | Ribonuclease Z | 1,200 | 966 | 964 | 970 | 769 |
| 3.1.3.15 | Histidinol-phosphatase | 209 | 227 | 230 | 237 | 404 |
| 3.1.3.25 | Inositol-phosphate phosphatase | 159 | 277 | 99 | 315 | 151 |
| 3.1.3.45 | 3-deoxy-manno-octulosonate-8-phosphatase | 5 | 8 | 13 | 3 | 1 |
| 3.1.4.46 | Glycerophosphodiester phosphodiesterase | 187 | 305 | 89 | 437 | 149 |
| 3.1.6.1 | Arylsulfatase | 280 | 277 | 316 | 289 | 521 |
| 3.11.1.1 | Phosphonoacetaldehyde hydrolase | 1,391 | 1,004 | 1,419 | 984 | 1,343 |
| 3.2.1.131 | Xylan alpha-1,2-glucuronosidase | 170 | 328 | 84 | 423 | 138 |
| 3.2.1.135 | Neopullulanase | 9 | 22 | 4 | 22 | 9 |
| 3.2.1.139 | Alpha-glucuronidase | 277 | 490 | 131 | 599 | 220 |
| 3.2.1.151 | Xyloglucan-specific endo-beta-1,4-glucanase | 673 | 1,231 | 309 | 1,450 | 521 |
| 3.2.1.169 | Protein O-GlcNAcase | 20 | 9 | 23 | 18 | 59 |
| 3.2.1.177 | Alpha-D-xyloside xylohydrolase | 311 | 565 | 127 | 699 | 231 |
| 3.2.1.190, 3.2.1.40 | Dioscin glycosidase (3-O-beta-D-Glc-diosgenin-forming)\|Alpha-L-rhamnosidase | 171 | 302 | 175 | 256 | 86 |
| 3.2.1.21 | Beta-glucosidase | 1,507 | 2,466 | 820 | 3,002 | 1,363 |
| 3.2.1.3 | Glucan 1,4-alpha-glucosidase | 108 | 201 | 112 | 159 | 47 |
| 3.2.1.37 | Xylan 1,4-beta-xylosidase | 233 | 464 | 142 | 586 | 205 |
| 3.2.1.4 | Cellulase | 708 | 1,254 | 307 | 1,526 | 578 |
| 3.2.1.40 | Alpha-L-rhamnosidase | 171 | 302 | 175 | 256 | 86 |
| 3.2.1.40, 3.2.1.66 | Alpha-L-rhamnosidase\|Quercitrinase | 171 | 302 | 175 | 256 | 86 |
| 3.2.1.49 | Alpha-N-acetylgalactosaminidase | 28 | 17 | 30 | 24 | 50 |
| 3.2.1.55 | Non-reducing end alpha-L-arabinofuranosidase | 1,216 | 2,208 | 610 | 2,772 | 987 |
| 3.2.1.6 | Endo-1,3(4)-beta-glucanase | 79 | 208 | 40 | 164 | 70 |
| 3.2.1.65 | Levanase | 325 | 596 | 195 | 747 | 319 |
| 3.2.1.8 | Endo-1,4-beta-xylanase | 663 | 1,279 | 610 | 1,922 | 617 |
| 3.2.1.89 | Arabinogalactan endo-beta-1,4-galactanase | 1,050 | 1,137 | 1,418 | 1,398 | 1,658 |
| 3.2.1.99 | Arabinan endo-1,5-alpha-L-arabinosidase | 541 | 854 | 542 | 722 | 264 |
| 3.2.2.9 | Adenosylhomocysteine nucleosidase | 650 | 746 | 758 | 881 | 675 |
| 3.4.11.4 | Tripeptide aminopeptidase | 919 | 760 | 790 | 731 | 723 |
| 3.4.11.9 | Xaa-Pro aminopeptidase | 194 | 350 | 180 | 344 | 122 |
| 3.4.13.22 | D-Ala-D-Ala dipeptidase | 1,150 | 1,168 | 1,585 | 1,511 | 1,772 |
| 3.4.14.4 | Dipeptidyl-peptidase III | 18 | 21 | 49 | 52 | 29 |
| 3.4.15.5 | Peptidyl-dipeptidase Dcp | 14 | 24 | 31 | 28 | 11 |
| 3.4.16.4 | Serine-type D-Ala-D-Ala carboxypeptidase | 678 | 307 | 712 | 576 | 1,576 |
| 3.4.21.102 | C-terminal processing peptidase | 855 | 1,036 | 890 | 1,166 | 1,251 |
| 3.4.21.53 | Endopeptidase La | 1,185 | 855 | 1,015 | 832 | 1,081 |
| 3.4.22.40 | Bleomycin hydrolase | 10 | 13 | 29 | 37 | 19 |
| 3.4.23.36 | Signal peptidase II | 1,397 | 1,343 | 1,572 | 1,302 | 1,356 |
| 3.4.24.71 | Endothelin-converting enzyme 1 | 9 | 12 | 22 | 21 | 5 |
| 3.5.1.10 | Formyltetrahydrofolate deformylase | 480 | 557 | 656 | 704 | 777 |
| **3.5.1.11** | **Penicillin amidase** | **313** | **480** | **190** | **494** | **162** |
| 3.5.1.2, 3.5.1.38 | Glutaminase\|Glutamin-(asparagin-)ase | 2,311 | 2,504 | 2,531 | 3,057 | 2,661 |
| 3.5.1.24 | Choloylglycine hydrolase | 311 | 389 | 185 | 494 | 162 |
| 3.5.2.3 | Dihydroorotase | 1,353 | 1,448 | 1,355 | 1,580 | 1,425 |
| 3.5.2.6 | Beta-lactamase | 159 | 258 | 600 | 433 | 85 |
| 3.5.2.7 | Imidazolonepropionase | 739 | 710 | 638 | 772 | 223 |
| 3.5.4.10 | IMP cyclohydrolase | 367 | 408 | 402 | 412 | 596 |
| 3.5.4.19 | Phosphoribosyl-AMP cyclohydrolase | 425 | 433 | 390 | 402 | 517 |
| 3.5.4.2 | Adenine deaminase | 523 | 189 | 199 | 35 | 20 |
| 3.5.4.25 | GTP cyclohydrolase II | 1,032 | 718 | 842 | 769 | 1,018 |
| 3.5.4.26 | Diaminohydroxyphosphoribosylaminopyrimidine deaminase | 82 | 104 | 110 | 116 | 153 |
| 3.5.4.28 | S-adenosylhomocysteine deaminase | 474 | 344 | 331 | 94 | 35 |
| 3.5.4.31 | S-methyl-5'-thioadenosine deaminase | 474 | 344 | 331 | 94 | 35 |
| 3.5.4.33 | tRNA(adenine(34)) deaminase | 1,508 | 1,418 | 1,551 | 1,303 | 1,470 |
| 3.6.1.27 | Undecaprenyl-diphosphate phosphatase | 1,394 | 1,311 | 1,560 | 1,338 | 1,239 |
| 3.6.1.31 | Phosphoribosyl-ATP diphosphatase | 295 | 363 | 359 | 368 | 517 |
| 3.6.3.12 | Potassium-transporting ATPase | 1,004 | 1,120 | 1,234 | 986 | 972 |
| 3.6.3.15 | Sodium-transporting two-sector ATPase | 2 | 2 | 3 | 2 | 2 |
| 3.6.3.25 | Sulfate-transporting ATPase | 274 | 481 | 422 | 208 | 236 |
| 3.6.3.28 | Phosphonate-transporting ATPase | 47 | 35 | 45 | 33 | 45 |
| 3.6.3.31 | Polyamine-transporting ATPase | 587 | 665 | 511 | 486 | 794 |
| 3.6.3.34 | Iron-chelate-transporting ATPase | 17 | 28 | 11 | 31 | 21 |
| 3.6.3.41 | Heme-transporting ATPase | 5 | 3 | 4 | 4 | 3 |
| 4.1.1.11 | Aspartate 1-decarboxylase | 1,192 | 1,061 | 1,305 | 1,140 | 1,182 |
| 4.1.1.19 | Arginine decarboxylase | 634 | 689 | 855 | 957 | 1,096 |
| 4.1.1.20 | Diaminopimelate decarboxylase | 1,212 | 888 | 1,171 | 1,099 | 1,165 |
| 4.1.1.37 | Uroporphyrinogen decarboxylase | 113 | 280 | 65 | 355 | 121 |
| 4.1.1.48 | Indole-3-glycerol-phosphate synthase | 863 | 647 | 693 | 752 | 1,018 |
| 4.1.1.49 | Phosphoenolpyruvate carboxykinase (ATP) | 1,794 | 1,720 | 1,792 | 1,404 | 1,552 |
| 4.1.2.19 | Rhamnulose-1-phosphate aldolase | 642 | 959 | 750 | 784 | 967 |
| 4.1.2.48, 4.1.2.5 | Low-specificity L-threonine aldolase\|L-threonine aldolase | 0 | 5 | 12 | 18 | 1 |
| 4.1.3.27 | Anthranilate synthase | 441 | 512 | 587 | 554 | 750 |
| 4.1.3.3 | N-acetylneuraminate lyase | 252 | 354 | 180 | 422 | 350 |
| 4.1.3.36 | 1,4-dihydroxy-2-naphthoyl-CoA synthase | 450 | 371 | 394 | 338 | 455 |
| 4.1.99.12 | 3,4-dihydroxy-2-butanone-4-phosphate synthase | 988 | 756 | 1,033 | 1,082 | 1,105 |
| 4.1.99.17 | Phosphomethylpyrimidine synthase | 1,113 | 771 | 975 | 823 | 1,018 |
| 4.2.1.10 | 3-dehydroquinate dehydratase | 2,063 | 1,939 | 1,840 | 1,821 | 1,957 |
| 4.2.1.113 | o-succinylbenzoate synthase | 898 | 725 | 932 | 606 | 1,108 |
| **4.2.1.126** | **N-acetylmuramic acid 6-phosphate etherase** | **1,194** | **955** | **1,140** | **1,010** | **1,508** |
| 4.2.1.19 | Imidazoleglycerol-phosphate dehydratase | 782 | 481 | 469 | 386 | 424 |
| 4.2.1.2 | Fumarate hydratase | 393 | 217 | 231 | 58 | 30 |
| 4.2.1.20 | Tryptophan synthase | 2,881 | 2,255 | 2,605 | 2,366 | 2,678 |
| 4.2.1.46 | dTDP-glucose 4,6-dehydratase | 1,686 | 1,938 | 1,483 | 1,881 | 1,494 |
| 4.2.1.49 | Urocanate hydratase | 884 | 892 | 663 | 1,246 | 349 |
| 4.2.1.7 | Altronate dehydratase | 14 | 17 | 19 | 20 | 30 |
| 4.2.1.8 | Mannonate dehydratase | 2,807 | 2,885 | 2,753 | 3,207 | 3,199 |
| 4.2.1.9 | Dihydroxy-acid dehydratase | 1,291 | 1,257 | 1,185 | 901 | 1,007 |
| 4.2.2.21 | Chondroitin-sulfate-ABC exolyase | 14 | 13 | 17 | 14 | 31 |
| 4.2.3.3 | Methylglyoxal synthase | 1,665 | 1,775 | 1,970 | 1,304 | 1,213 |
| 4.2.3.5 | Chorismate synthase | 1,086 | 934 | 1,337 | 1,351 | 882 |
| 4.3.1.1 | Aspartate ammonia-lyase | 116 | 115 | 117 | 125 | 183 |
| 4.3.1.3 | Histidine ammonia-lyase | 615 | 610 | 682 | 873 | 168 |
| 4.3.1.4 | Formimidoyltetrahydrofolate cyclodeaminase | 19 | 31 | 30 | 10 | 5 |
| 4.3.2.1 | Argininosuccinate lyase | 1,614 | 1,346 | 1,285 | 1,306 | 1,306 |
| 4.3.3.7 | 4-hydroxy-tetrahydrodipicolinate synthase | 1,364 | 1,224 | 1,251 | 948 | 1,353 |
| 4.4.1.11 | Methionine gamma-lyase | 2 | 1 | 3 | 2 | 3 |
| 4.4.1.21 | S-ribosylhomocysteine lyase | 116 | 157 | 342 | 549 | 106 |
| **4.6.1.12** | **2-C-methyl-D-erythritol 2,4-cyclodiphosphate synthase** | **1,495** | **1,298** | **1,328** | **1,069** | **1,257** |
| 5.1.1.20 | L-Ala-D/L-Glu epimerase | 383 | 412 | 386 | 454 | 711 |
| 5.1.1.3 | Glutamate racemase | 1,665 | 1,418 | 1,687 | 1,589 | 1,916 |
| 5.1.1.7 | Diaminopimelate epimerase | 1,443 | 1,179 | 1,494 | 1,101 | 1,658 |
| 5.1.3.11 | Cellobiose epimerase | 1,043 | 979 | 1,018 | 1,466 | 399 |
| 5.1.3.12 | UDP-glucuronate 5'-epimerase | 12 | 9 | 15 | 13 | 34 |
| 5.1.3.13 | dTDP-4-dehydrorhamnose 3,5-epimerase | 309 | 352 | 261 | 316 | 272 |
| 5.1.3.14 | UDP-N-acetylglucosamine 2-epimerase (non-hydrolyzing) | 1,588 | 1,064 | 1,303 | 1,000 | 1,678 |
| 5.1.3.32 | L-rhamnose mutarotase | 257 | 483 | 221 | 528 | 191 |
| 5.1.3.8 | N-acylglucosamine 2-epimerase | 646 | 1,115 | 572 | 1,092 | 358 |
| 5.1.3.9 | N-acylglucosamine-6-phosphate 2-epimerase | 636 | 310 | 197 | 114 | 17 |
| 5.3.1.12 | Glucuronate isomerase | 1,094 | 1,086 | 1,027 | 1,225 | 1,178 |
| 5.3.1.13 | Arabinose-5-phosphate isomerase | 494 | 657 | 530 | 763 | 699 |
| **5.3.1.14** | **L-rhamnose isomerase** | **1,631** | **1,616** | **1,857** | **1,698** | **1,776** |
| 5.3.1.16 | isomerase | 748 | 598 | 743 | 561 | 730 |
| 5.3.1.17 | 5-dehydro-4-deoxy-D-glucuronate isomerase | 1,155 | 1,257 | 1,444 | 1,364 | 1,741 |
| 5.3.1.24 | Phosphoribosylanthranilate isomerase | 1,102 | 792 | 1,057 | 722 | 1,389 |
| 5.3.1.25 | L-fucose isomerase | 696 | 786 | 1,178 | 1,167 | 1,144 |
| 5.3.1.28 | D-sedoheptulose 7-phosphate isomerase | 33 | 47 | 285 | 275 | 38 |
| 5.3.1.4 | L-arabinose isomerase | 672 | 993 | 958 | 911 | 1,016 |
| 5.3.1.5 | Xylose isomerase | 969 | 811 | 928 | 830 | 1,025 |
| 5.3.1.6 | Ribose-5-phosphate isomerase | 1,363 | 1,503 | 1,224 | 1,856 | 1,846 |
| 5.4.2.10 | Phosphoglucosamine mutase | 1,188 | 1,358 | 1,186 | 1,128 | 1,280 |
| 5.4.3.2 | Lysine 2,3-aminomutase | 21 | 34 | 35 | 30 | 27 |
| 5.4.4.2 | Isochorismate synthase | 67 | 127 | 47 | 183 | 61 |
| 5.4.99.18 | 5-(carboxyamino)imidazole ribonucleotide mutase | 1,606 | 1,586 | 1,685 | 1,496 | 1,751 |
| 5.4.99.2 | Methylmalonyl-CoA mutase | 1,488 | 1,576 | 2,122 | 2,001 | 2,111 |
| 5.4.99.25 | tRNA pseudouridine(55) synthase | 1,603 | 1,540 | 1,832 | 1,602 | 1,663 |
| 5.4.99.5 | Chorismate mutase | 74 | 91 | 130 | 190 | 119 |
| 5.4.99.9 | UDP-galactopyranose mutase | 326 | 275 | 344 | 217 | 988 |
| 6.1.1.12 | Aspartate--tRNA ligase | 681 | 517 | 559 | 377 | 622 |
| 6.1.1.18 | Glutamine--tRNA ligase | 910 | 789 | 808 | 590 | 651 |
| 6.1.1.19 | Arginine--tRNA ligase | 1,167 | 972 | 1,066 | 812 | 765 |
| 6.1.1.21 | Histidine--tRNA ligase | 945 | 886 | 814 | 782 | 871 |
| 6.1.1.6 | Lysine--tRNA ligase | 1,844 | 1,630 | 1,667 | 1,494 | 1,625 |
| 6.1.1.9 | Valine--tRNA ligase | 1,222 | 1,050 | 1,107 | 850 | 788 |
| 6.2.1.26 | o-succinylbenzoate--CoA ligase | 90 | 133 | 73 | 195 | 107 |
| 6.2.1.30 | Phenylacetate--CoA ligase | 1,677 | 1,220 | 1,712 | 1,590 | 2,114 |
| 6.3.1.1 | Aspartate--ammonia ligase | 1,117 | 822 | 952 | 811 | 907 |
| 6.3.1.5 | NAD(+) synthase | 53 | 102 | 101 | 80 | 59 |
| 6.3.2.1 | Pantoate--beta-alanine ligase (AMP-forming) | 1,400 | 1,191 | 1,387 | 1,114 | 864 |
| 6.3.2.10 | UDP-N-acetylmuramoyl-tripeptide--D-alanyl-D-alanine ligase | 1,515 | 1,187 | 1,664 | 1,151 | 1,533 |
| 6.3.2.13 | UDP-N-acetylmuramoyl-L-alanyl-D-glutamate--2,6-diaminopimelate ligase | 560 | 289 | 301 | 166 | 198 |
| 6.3.2.17 | Tetrahydrofolate synthase | 6 | 14 | 12 | 6 | 2 |
| 6.3.2.4 | D-alanine--D-alanine ligase | 932 | 786 | 927 | 698 | 550 |
| 6.3.2.8 | UDP-N-acetylmuramate--L-alanine ligase | 1,347 | 1,183 | 1,345 | 1,128 | 1,115 |
| 6.3.3.3 | Dethiobiotin synthase | 1,519 | 1,102 | 1,334 | 1,480 | 2,044 |
| 6.3.4.14 | Biotin carboxylase | 484 | 536 | 544 | 627 | 852 |
| 6.3.4.2 | CTP synthase (glutamine hydrolyzing) | 1,126 | 1,197 | 1,189 | 1,175 | 1,207 |
| 6.3.4.20 | 7-cyano-7-deazaguanine synthase | 1,335 | 958 | 1,277 | 1,103 | 1,390 |
| 6.3.4.21 | Nicotinate phosphoribosyltransferase | 639 | 862 | 756 | 852 | 518 |
| 6.3.4.3 | Formate--tetrahydrofolate ligase | 2,827 | 2,919 | 3,221 | 3,361 | 2,655 |
| 6.3.4.6 | Urea carboxylase | 225 | 304 | 210 | 226 | 232 |
| 6.3.5.1 | NAD(+) synthase (glutamine-hydrolyzing) | 17 | 36 | 43 | 11 | 2 |
| 6.3.5.4 | Asparagine synthase (glutamine-hydrolyzing) | 163 | 584 | 93 | 57 | 79 |
| 6.3.5.5 | Carbamoyl-phosphate synthase (glutamine-hydrolyzing) | 3,315 | 3,025 | 3,262 | 2,689 | 2,877 |
| 6.3.5.9 | Hydrogenobyrinic acid a,c-diamide synthase (glutamine-hydrolyzing) | 2 | 3 | 8 | 19 | 3 |
| 6.4.1.3 | Propionyl-CoA carboxylase | 61 | 82 | 79 | 30 | 18 |
| 6.5.1.2, 6.5.1.6 | DNA ligase (NAD(+))\|DNA ligase (ATP or NAD(+)) | 1,005 | 871 | 928 | 692 | 674 |

Table S5. **Changes in the relative abundance of amino acids in stool, cecal content and liver in GF-MCD-B mice compared to GF-MCD mice.** Significant differences in the relative abundance of each amino acid between GF-MCD and GF-MCD-B were assessed by Mann-Whitney test. For those amino acids showing significant changes in stool, abundance changes were also assessed in cecal content and liver. FC: fold change (GF-MCD-B relative to GF-MCD); NC: not changed significantly.

|  | **Stool** | |  | **Cecal content** | |  | **Liver** | |
| --- | --- | --- | --- | --- | --- | --- | --- | --- |
| **Amino acid** | **FC** | ***p*** |  | **FC** | ***p*** |  | **FC** | ***p*** |
| Homocysteine | 93.80 | <0.001 |  | 76.57 | <0.001 |  | 2.96 | 0.020 |
| S-adenosylhomocysteine (SAH) | 75.73 | <0.001 |  | 26.85 | <0.001 |  | 1.91 | 0.016 |
| Cysteine/serine ratio | 25.92 | <0.001 |  | 38.85 | <0.001 |  | NC |  |
| Cysteine | 14.93 | <0.001 |  | 11.58 | <0.001 |  | NC |  |
| Taurine | 3.63 | <0.001 |  | 2.65 | <0.001 |  | NC |  |
| Acetylcholine | 2.89 | 0.001 |  | 2.88 | <0.001 |  | NC |  |
| Creatinine | 1.37 | 0.020 |  | NC |  |  | NC |  |
| Serine | -2.32 | 0.010 |  | -3.54 | <0.001 |  | NC |  |
| Arginine | -2.33 | 0.029 |  | -1.22 | 0.010 |  | NC |  |
| Phenylalaine | -2.41 | 0.006 |  | -3.20 | <0.001 |  | 1.48 | 0.029 |
| Tyrosine | -2.41 | 0.010 |  | -2.47 | <0.001 |  | NC |  |
| Methionine Sulfoxide | -2.46 | 0.002 |  | -3.49 | <0.001 |  | NC |  |
| Cystine | -2.47 | 0.036 |  | -2.24 | 0.006 |  | NC |  |
| Glutamine | -2.77 | 0.001 |  | -2.69 | <0.001 |  | 1.64 | 0.004 |
| Aspartic acid | -3.19 | 0.002 |  | -3.06 | <0.001 |  | NC |  |
| Isoleucine | -3.49 | <0.001 |  | -3.91 | <0.001 |  | NC |  |
| Leucine | -4.02 | <0.001 |  | -3.38 | 0.003 |  | NC |  |
| Threonine | -8.06 | <0.001 |  | -9.72 | <0.001 |  | NC |  |
| Asparagine | -12.59 | <0.001 |  | -44.55 | <0.001 |  | 1.22 | 0.043 |
| Glutamic acid | NC |  |  | 1.85 | 0.006 |  | NC |  |
| Choline | NC |  |  | 1.26 | 0.002 |  | NC |  |
| Histidine | NC |  |  | -1.73 | 0.001 |  | NC |  |
| Lysine | NC |  |  | -2.16 | <0.001 |  | NC |  |
| Valine | NC |  |  | -3.05 | <0.001 |  | NC |  |
| Glycine | NC |  |  | NC |  |  | 1.79 | 0.010 |
| Tryptophan | NC |  |  | -2.54 | <0.001 |  | 1.49 | 0.010 |
| Kynurenine | NC |  |  | NC |  |  | -1.66 | 0.036 |
| Kynurenine/tryptophan ratio | NC |  |  | 2.65 | 0.001 |  | -2.73 | 0.001 |
| Carnitine | NC |  |  | NC |  |  | -1.23 | 0.024 |
| Hypotaurine | NC |  |  | NC |  |  | -1.53 | 0.010 |
| 4-hydroxyproline | NC |  |  | NC |  |  | NC |  |
| Alanine | NC |  |  | NC |  |  | NC |  |
| Citrulline | NC |  |  | NC |  |  | NC |  |
| Creatine | NC |  |  | NC |  |  | NC |  |
| Methionine | NC |  |  | NC |  |  | NC |  |
| Methionine Sulfone | NC |  |  | NC |  |  | NC |  |
| Ornithine | NC |  |  | NC |  |  | NC |  |
| Proline | NC |  |  | NC |  |  | NC |  |
| Sarcosine | NC |  |  | NC |  |  | NC |  |

Table S6. **Selection of strains for the synthetic consortium, and gene copy numbers of selected amino acid-metabolizing enzymes in colonizing bacterial species.** All strains detected in the human cohort, and belonging to the eight species of the bacterial consortium are shown. For the six colonizing species, raw genome sequencing files were retrieved from the NCBI data hub, and annotated with the prokka pipeline, available on the Proksee online tool, to determine the presence of the selected enzymes in each bacterial strain genome. Genome sequences were not available for unclassified strains, nor for *Bacteroides ovatus* CL02T12C04. Strains are sorted by detection rate in the 340 human study participants. For each species, the most abundant strain commercially available (shown in bold) was selected for the *in vivo* study.

|  |  | **Detection rate** | **Mean abundance** | **NCBI Genome accession number** | **Serine acetyl-transferase (EC 2.3.1.30)** | **Cysteine synthase**  **(EC 2.5.1.47)** | **Asparaginase (EC 3.5.1.1)** |  |
| --- | --- | --- | --- | --- | --- | --- | --- | --- |
| *Bacteroides caccae* | | 76% | 0.19% |  |  |  |  | |
|  | ***Bacteroides caccae* ATCC 43185** | 51% | 0.16% | GCF_000169015.1 | 0 | 2 | 3 |  |
|  | *Bacteroides caccae* CL03T12C61 | 19% | 0.03% | GCF_018292205.1 | 0 | 2 | 3 |  |
|  | *Bacteroides caccae* (unclassified) | 6% | 0.00% | - | - | - | - |  |
| *Bacteroides finegoldii* | | 61% | 0.22% |  |  |  |  | |
|  | ***Bacteroides finegoldii* DSM 17565** | 55% | 0.21% | GCF_000156195.1 | 0 | 2 | 3 |  |
|  | *Bacteroides finegoldii* CL09T03C10 | 6% | 0.00% | GCF_000304195.1 | 0 | 3 | 3 |  |
| *Bacteroides ovatus* | | 94% | 0.63% |  |  |  |  | |
|  | *Bacteroides ovatus* SD CMC 3f | 46% | 0.28% | GCF_000178275.1 | 0 | 2 | 3 |  |
|  | *Bacteroides ovatus* CL02T12C04 | 33% | 0.23% | - | - | - | - |  |
|  | ***Bacteroides ovatus* ATCC 8483** | 6% | 0.03% | GCF_000154125.1 | 0 | 2 | 3 |  |
|  | *Bacteroides ovatus* 3_8_47FAA | 4% | 0.04% | GCF_000218325.1 | 1 | 2 | 3 |  |
|  | *Bacteroides ovatus* CL03T12C18 | 2% | 0.03% | GCF_018492845.1 | 0 | 3 | 3 |  |
|  | *Bacteroides ovatus* str. 3725 D1 iv | 2% | 0.01% | GCF_000699725.1 | 1 | 3 | 3 |  |
|  | *Bacteroides ovatus* str. 3725 D9 iii | 1% | 0.00% | GCF_000699665.1 | 1 | 2 | 3 |  |
| *Bacteroides uniformis* | | 92% | 2.24% |  |  |  |  | |
|  | ***Bacteroides uniformis* ATCC 8492** | 40% | 0.88% | GCF_900107315.1 | 1 | 2 | 2 |  |
|  | *Bacteroides uniformis* (unclassified) | 19% | 0.39% | - | - | - | - |  |
|  | *Bacteroides uniformis* str. 3978 T3 ii | 14% | 0.43% | GCF_000699825.1 | 0 | 2 | 2 |  |
|  | *Bacteroides uniformis* str. 3978 T3 i | 8% | 0.28% | GCF_000699885.1 | 0 | 3 | 2 |  |
|  | *Bacteroides uniformis* CL03T12C37 | 6% | 0.11% | GCF_018292165.1 | 0 | 2 | 2 |  |
|  | *Bacteroides uniformis* dnLKV2 | 6% | 0.15% | GCF_000403175.1 | 1 | 2 | 2 |  |
| *Parabacteroides distasonis* | | 84% | 0.49% |  |  |  |  | |
|  | *Parabacteroides distasonis* (unclassified) | 27% | 0.13% | - | - | - | - |  |
|  | *Parabacteroides distasonis* CL09T03C24 | 26% | 0.17% | GCF_000307435.1 | 0 | 1 | 1 |  |
|  | ***Parabacteroides distasonis* ATCC 8503** | 13% | 0.06% | GCF_000012845.1 | 0 | 1 | 1 |  |
|  | *Parabacteroides distasonis* CL03T12C09 | 8% | 0.08% | GCF_018292145.1 | 0 | 1 | 1 |  |
|  | *Parabacteroides distasonis* str. 3999B T(B) 6 | 5% | 0.03% | GCF_000699765.1 | 0 | 1 | 1 |  |
|  | *Parabacteroides distasonis* str. 3999B T(B) 4 | 2% | 0.01% | GCF_000699905.1 | 0 | 1 | 1 |  |
|  | *Parabacteroides distasonis* str. 3776 Po2 i | 2% | 0.01% | GCF_000699745.1 | 0 | 1 | 1 |  |
|  | *Parabacteroides distasonis* str. 3776 D15 i | 0% | 0.00% | GCF_000699785.1 | 0 | 1 | 1 |  |
|  | *Parabacteroides distasonis* str. 3776 D15 iv | 0% | 0.00% | GCF_000699805.1 | 0 | 1 | 1 |  |
| *Alistipes onderdonkii* | | 66% | 0.36% |  |  |  |  | |
|  | ***Alistipes onderdonkii subsp. vulgaris*** | 35% | 0.26% | GCF_006542645.1 | 0 | 1 | 1 |  |
|  | *Alistipes onderdonkii* DSM 19147 | 31% | 0.09% | GCF_000374505.1 | 0 | 1 | 1 |  |
| *Anaerobutyricum hallii* | | 91% | 1.12% |  |  |  |  |  |
|  | ***Anaerobutyricum hallii* ATCC 27751** | 91% | 1.12% |  |  |  |  |  |
| *Gemmiger formicilis* | | 91% | 1.04% |  |  |  |  |  |
|  | ***Gemmiger formicilis* ATCC 27749** | 91% | 1.04% |  |  |  |  |  |

# SUPPLEMENTARY FIGURES

**
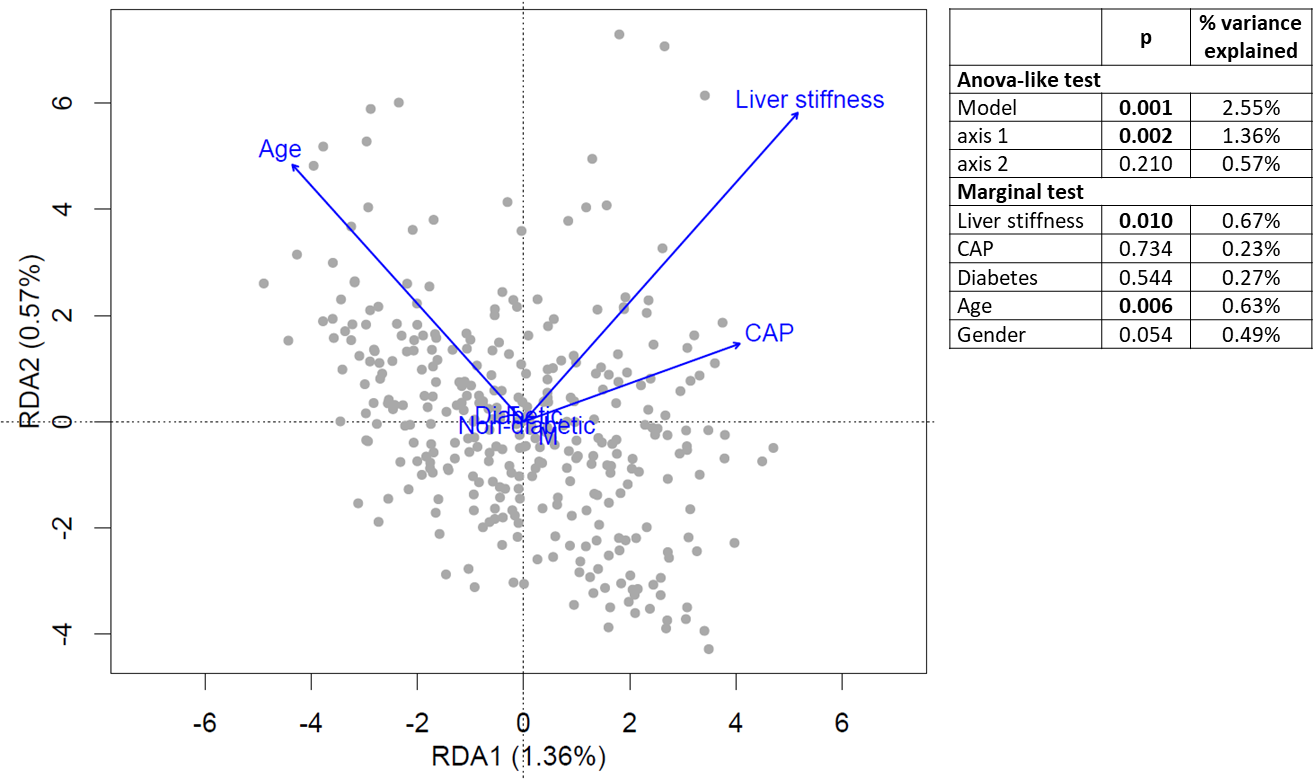
**

Figure S1. **The contribution of clinical parameters to variation in the liver fibrosis-associated microbiome signature.** To determine the confounding effects of common comorbidities on the identified liver fibrosis-associated microbiome signature (Figure 1c-d), redundancy analysis was performed with FibroScan liver stiffness, FibroScan CAP, diabetes, age and gender as explanatory variables, and relative abundance of the 20 species associated with liver fibrosis and/or advanced liver fibrosis as response variables. ANOVA-like significance tests of the model, axes and explanatory variables were performed to determine which of these clinical variables contributed significantly to abundance variation of the 20 species across samples.


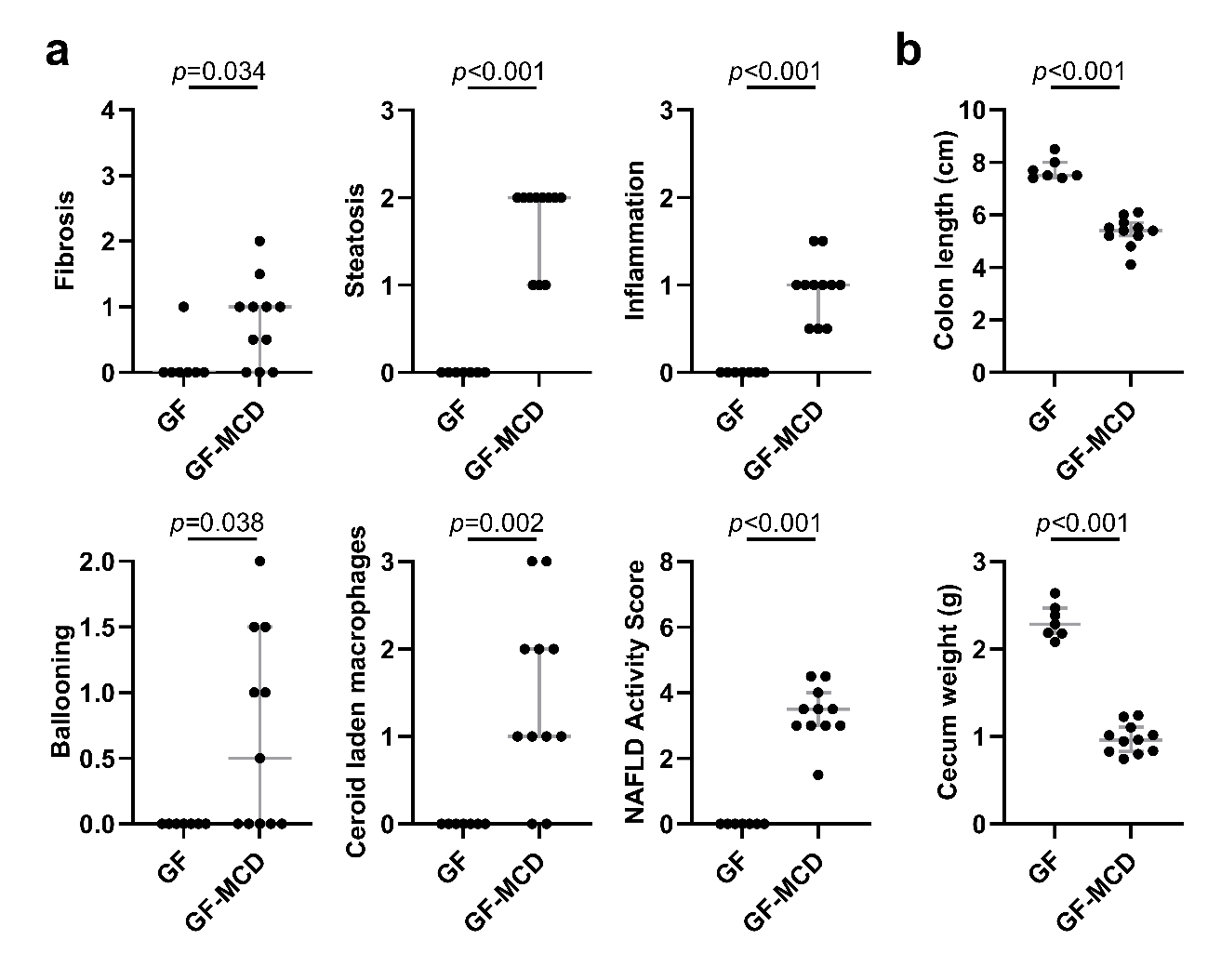


Figure S2. **Induction of steatohepatitis and liver fibrosis by methionine- and choline-deficient diet (MCD) in germ-free (GF)** **mice.** Liver histology (a) and necropsy measurements (b) of GF mice under regular chow (GF, n=7), and of GF mice after 6 weeks of MCD (GF-MCD, n=11). Error bars show median with interquartile range. Significance was assessed by the Mann-Whitney *U* test.


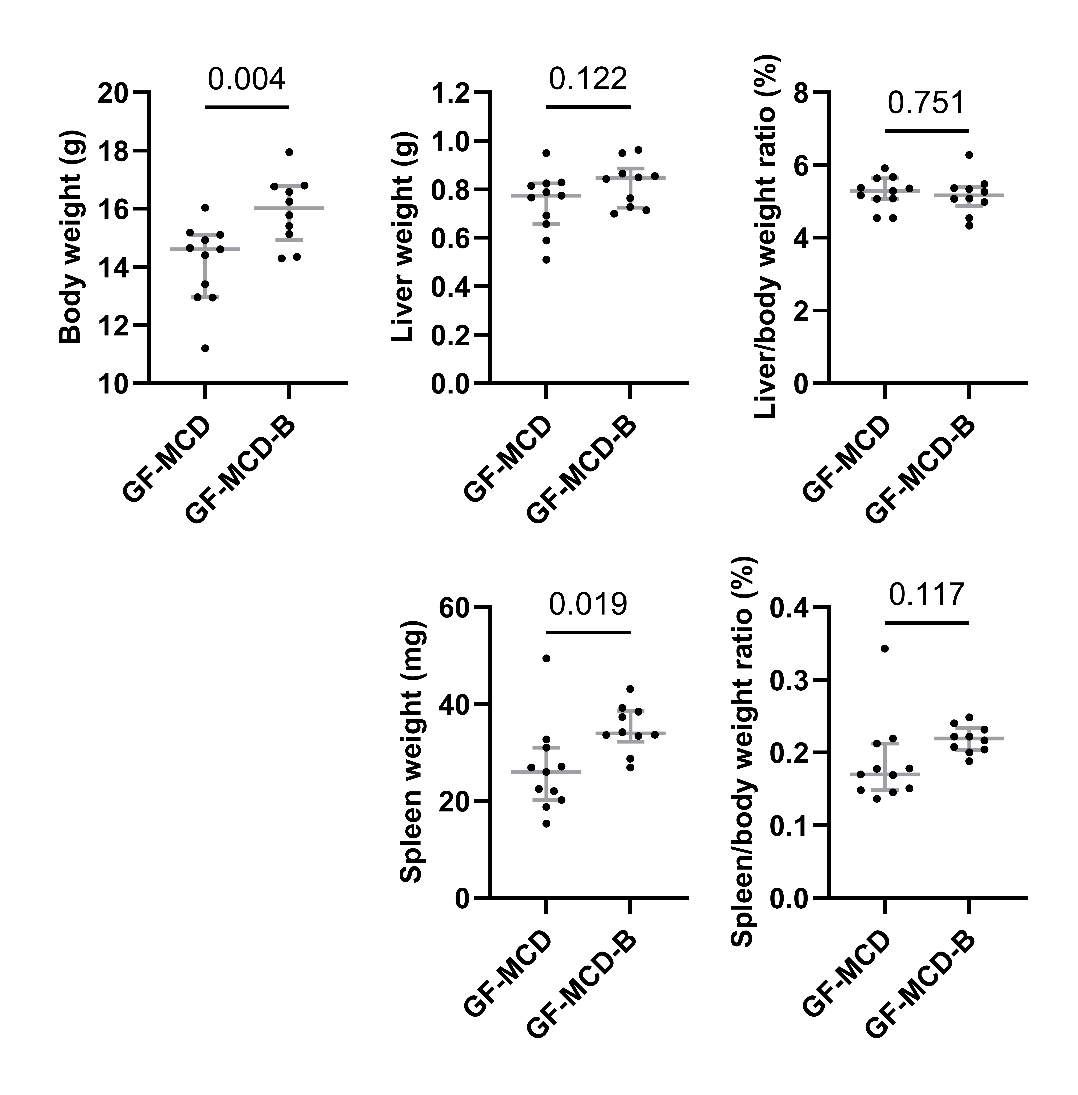


Figure S3. **Body weight, liver weight and spleen weight of GF-MCD and GF-MCD-B mice at time of necropsy.**
